# Longitudinal DNA methylation analysis of adult-type *IDH*-mutant gliomas

**DOI:** 10.1186/s40478-023-01520-1

**Published:** 2023-02-04

**Authors:** Sandra Ferreyra Vega, Thomas Olsson Bontell, Teresia Kling, Asgeir Store Jakola, Helena Carén

**Affiliations:** 1 Department of Clinical Neuroscience, Institute of Neuroscience and Physiology, Sahlgrenska Academy, University of Gothenburg, Blå Stråket 7, 413 45 Gothenburg, Sweden; 2 Sahlgrenska Center for Cancer Research, Department of Medical Biochemistry and Cell Biology, Institute of Biomedicine, Sahlgrenska Academy, University of Gothenburg, Gothenburg, Sweden; 3 Department of Physiology, Institute of Neuroscience and Physiology, Sahlgrenska Academy, University of Gothenburg, Gothenburg, Sweden; 4 Department of Clinical Pathology, Sahlgrenska University Hospital, Gothenburg, Sweden; 5 Department of Neurosurgery, Sahlgrenska University Hospital, Gothenburg, Sweden; 6 Department of Neurosurgery, St. Olavs University Hospital, Trondheim, Norway

**Keywords:** *IDH*-mutant gliomas, DNA methylation profiling, Tumor recurrence, Longitudinal analysis

## Abstract

Diffuse gliomas are the most prevalent malignant primary brain tumors in adults and remain incurable despite standard therapy. Tumor recurrence is currently inevitable, which contributes to a persistent high morbidity and mortality in these patients. In this study, we examined the genome-wide DNA methylation profiles of primary and recurrent adult-type *IDH*-mutant gliomas to elucidate DNA methylation changes associated with tumor progression (with or without malignant transformation). We analyzed DNA methylation profiles of 37 primary *IDH*-mutant gliomas and 42 paired recurrences using the DNA methylation EPIC beadChip array. DNA methylation-based classification reflected the tumor progression over time. We observed a methylation subtype switch in a proportion of *IDH*-mutant astrocytomas; the primary tumors were subclassified as low-grade astrocytomas, which progressed to high-grade astrocytomas in the recurrent tumors. The CNS WHO grade 4 *IDH*-mutant astrocytomas did not always resemble methylation subclasses of higher grades. The number of differentially methylated CpG sites increased over time, and astrocytomas accumulated more differentially methylated CpG sites than oligodendrogliomas during tumor progression. Few differentially methylated CpG sites were shared between patients. We demonstrated that DNA methylation profiles are mostly maintained during *IDH*-mutant glioma progression, but CpG site-specific methylation alterations can occur.

**Supplementary Information:**

The online version contains supplementary material available at 10.1186/s40478-023-01520-1.

## Introduction

Diffuse gliomas are the most common aggressive primary brain neoplasms in adults characterized by an extensive infiltrative growth and heterogeneity between patients and within tumors [7, 11, 22, 24, 29, 31, 41]. Mutations in the *isocitrate dehydrogenase* genes 1 and 2 (*IDH1/IDH2*) define a glioma subtype associated with improved patient outcome in comparison to *IDH*-wildtype gliomas with molecular features of glioblastoma [16, 41]. In addition, *IDH*-mutant gliomas with deletions of chromosome arms 1p and 19q (1p/19q-codeleted), characterize the oligodendroglioma subtype with more favorable overall survival in comparison to *IDH*-mutant gliomas with retained 1p/19q (astrocytomas). Still, despite multidisciplinary treatment with surgery, radio- and chemotherapy, *IDH*-mutant gliomas recur and the tumors usually develop therapeutic resistance, making them incurable with current treatment [37].

Gliomas with *IDH1*/*2* mutations display a distinct methylation pattern characterized by DNA hypermethylation of CpG islands, the so called CpG island methylator phenotype (CIMP) [21, 34]. *IDH*-mutant gliomas with CIMP (G-CIMP) signatures are associated with a more favorable clinical outcome. Further refinement of G-CIMP based on the degree of DNA methylation (G-CIMP-high and G-CIMP-low) has revealed differences in prognosis where primary G-CIMP-low tumors exhibit lower degree of methylation and poorer clinical outcome compared to G-CIMP-high tumors [7]. In addition, G-CIMP-low tumors at recurrence have been shown to resemble *IDH*-wildtype glioblastomas [9].

We recently reported spatial methylation variability in *IDH*-mutant gliomas where we identified a high number of differentially methylated positions (DMPs) within the tumors as well as heterogeneous chromosomal copy number alterations (CNAs), with potential clinical implications in diagnostics [11]. We further observed that pediatric tumors of higher grade had more DMPs than tumors of lower grades [39]. As DNA methylation alterations also may occur and accumulate throughout progression of primary tumors in adults, it is therefore important to elucidate the dynamics of DNA methylation that may be associated with tumor progression. In this study, we profiled the longitudinal genome-wide DNA methylation patterns across 37 adult patients with *IDH*-mutant gliomas.

## Materials and methods

### Patient characteristics

A clinical consecutive cohort of 129 adult patients (≥ 18 years old) underwent surgery for primary *IDH*-mutant diffuse glioma between 2007 and 2016 at the neurosurgical department at the Sahlgrenska University Hospital (Gothenburg, Sweden). Treatment recommendations were discussed individually for each patient at the multidisciplinary neuro-oncological tumor board at the Sahlgrenska University Hospital. An initial selection criterion for this study was based on an independent research study of the primary tumors as previously described [10]. We further selected for patients reoperated for glioma recurrence (with or without malignant transformation), which accounted for 37% (48/129) of the entire *IDH*-mutant glioma cohort. Patients were excluded when formalin-fixed paraffin-embedded (FFPE) tumor tissue samples for subsequent molecular analyses were not available (n = 11). FFPE samples from the primary tumors and corresponding core clinical and molecular data (e.g. 2016 World Health Organization (WHO) diagnosis, age at diagnosis, survival and status of *IDH1*/*2* mutation and 1p/19q codeletion) were collected in our previous study [10]. FFPE tissue samples from the recurrent tumors were provided by the pathology department at the Sahlgrenska University Hospital. In total, we included 79 tumors from 37 patients comprising 32 patients with a primary tumor and a single matched recurrence and five patients with two tumor recurrences. The end of follow-up date of the patients was January 1st, 2022.

### DNA methylation array profiling

Isolation of genomic DNA from the FFPE tumor recurrences and subsequent generation of genome-wide DNA methylation array data was performed as described for the primary tumors [10]. Briefly, isolated DNA was bisulfite-treated with the EZ DNA Methylation Kit (Zymo Research, Orange, CA, USA) prior restoration with the Infinium HD FFPE DNA Restore Kit (Illumina Inc., San Diego, CA, USA). DNA methylation data was generated using the Infinium Illumina methylation EPIC beadChip array and analyzed with the statistical software R with R studio (version 4.1.2) [26]. The level of methylation for each CpG site in the EPIC array was denoted as a β-value. We estimated the proportion of neoplastic cells in the tumor samples based on methylation array data using the R package InfiniumPurify [25]. We applied the *getpurity()* function and selected the tumor type *LGG,* which is the reference methylation dataset for *brain lower-grade gliomas* from The Cancer Genome Atlas (TCGA) included in the package. The LGG dataset was applied to predict tumor purity scores for all samples in this study, although it might not be completely representative for certain tumor samples (e.g. high-grade gliomas).

Raw methylation array data (.idat files) were uploaded to the Molecular neuropathology (MNP) classifier version 12.5 (unpublished) developed for the central nervous system (CNS) tumors (https://www.molecularneuropathology.org/mnp) [19]. The classifier reported methylation class families with respective prediction estimates (calibrated scores) and we examined these as indicated by Capper et al. using the 0.90 threshold defined for accurate prediction and a score < 0.30 represented unclassifiable cases [5, 19]. The G-CIMP status was identified by hierarchical clustering of the most deviating 1000 CpG sites with G-CIMP positive and negative tumor samples from TCGA [21], and with glioblastoma *IDH*-wildtype samples exhibiting a G-CIMP negative signature from our previous study [38]. We further discriminated G-CIMP status into G-CIMP high/low with the methylation-based TCGA classifier for glioma subtyping according to Ceccarelli et al. [7] using the R package TCGAbiolinks [8]. The promotor methylation status of O6-methylguanine-DNA methyltransferase (*MGMT*) was predicted with the R package MGMTSTP27 [2, 3].

#### Differential methylation analysis

CpG sites located in regions with chromosomal homozygous deletions (CNA segment < − 0.4) were removed prior differential methylation analysis. We identified differentially methylated CpG positions (DMPs, Δβ ≥ 0.30) between primary and paired first recurrences per patient as previously described [38]. We also calculated DMPs (Δβ ≥ 0.20) between groups of samples, i.e. primary tumors versus first recurrences, with ChAMP [20, 33] and performed a Gene set enrichment analysis (GSEA) of these DMPs applying the GOmeth method as indicated [18].

#### Chromosomal copy number alteration analysis

We measured CNAs from raw methylation array data using the R package conumee [12]. CNA profiles were visually examined for detection of 1p/19q codeletion and homozygous deletion of *MGMT* and *CDKN2A*/*B*. Homozygous deletions were defined with a threshold ≤ − 0.40 [6, 28].

### Immunohistochemical analysis of IDH1 and ATRX

Immunohistochemical analysis of IDH1 (R132H) was carried out as previously described [11]. ATRX (α-thalassemia/mental-retardation-syndrome-X-linked) immunostaining was performed as for IDH1 immunostaining except that the EnVision FLEX + system was used. IDH1 (DIA-H09, Dianova, Hamburg, Germany) and ATRX (HPA001906, Sigma Aldrich, Saint Louis, MO, USA) were visualized with 3,3ʹ-diaminobenzidine (DAB) chromogen. The sections were counterstained with Mayer’s hematoxylin for nuclei localization.

### Assessment of 1p/19q codeletion and *CDKN2A/B* status by FISH

Status of 1p/19q and *CDKN2A*/*B* was assessed by Fluorescent In situ Hybridization (FISH) according to the protocols used for routine clinical detection of these markers. We used probes for 1p36/1q25 and 19p13/19q13 in the analysis of 1p/19q codeletion and 9p21/CEP9 for *CDKN2A/B* homozygous deletion.

### Reclassification of tumors according to 2021 WHO criteria

We updated the histopathological diagnoses of the patients to the current 2021 WHO classification system of CNS tumors as the diagnoses were established on the basis of previous classification criteria (2007 or 2016 WHO [15, 17]). We integrated histopathological information (tumor morphology and grade) and molecular data (*IDH* mutation, 1p/19q codeletion, ATRX and *CDKN2A*/*B* homozygous deletion) available at time of diagnosis and/or generated retrospectively in our facilities at the Sahlgrenska University Hospital and the Sahlgrenska Center for Cancer Research. Since we previously showed that the status of 1p/19q codeletion can accurately be determined for the primary tumors using methylation array data [10], we integrated 1p/19q codeletion status on the histopathological diagnoses of the recurrent tumors that did not have clinical determination of this marker at time of diagnosis (n = 35).

### Statistical analyses

The program R was used for statistical computing [26]. Wilcox two-sided t-test was used to evaluate statistical significance between groups. The R package corrplot [36] was used for Pearson’s correlation and the R packages survival [32] and survminer [14] were employed for estimation of overall survival probabilities. *P*-values resulting from DMP analysis and GSEA were adjusted for multiple comparisons with the Benjamini–Hoechberg method (significance *p*-value < 0.05).

## Results

### Molecular reclassification according to 2021 WHO criteria

This study comprised 37 patients with primary *IDH*-mutant gliomas and 42 paired recurrences out of which five of these occurred as second recurrences. Patients’ age at primary diagnosis ranged from 18 to 69 years, with a mean age of 38 years. The median time to first recurrence and median overall survival from initial diagnosis was 4 years and 7 years, respectively. The clinicopathological features of the patients are presented in Table 1.

**Table 1:** Clinical characteristics of the studied cohort of patients with primary diffuse *IDH*-mutant gliomas (n = 37)

Variables	Number of subjects (n = 37)
Gender	
Female	15 (40.5)
Ratio male:female	1.5
Age at diagnosis—years	
Mean ± SD	38.1 ± 11.5
Tumor location	
Frontal	23 (62.2)
Insular	2 (5.4)
Occipital	1 (2.7)
Parietal	5 (13.5)
Temporal	6 (16.2)
Recurrence location in relation to primary tumor	
Local	36 (97.3)
Distant	1 (2.7)
Histomolecular reclassification of primary tumors (2021 WHO CNS criteria)	
Astrocytoma, *IDH*-mutant CNS WHO grade 2	12 (32.4)
Astrocytoma, *IDH*-mutant CNS WHO grade 3	11 (29.7)
Astrocytoma, *IDH*-mutant CNS WHO grade 4	2 (5.4)
Oligodendroglioma, *IDH*-mutant and 1p/19q-codeleted, CNS WHO grade 2	10 (27.0)
Oligodendroglioma, *IDH*-mutant and 1p/19q-codeleted, CNS WHO grade 3	2 (5.4)

*SD* standard deviation, *CNS* central nervous system

The diagnoses of the patients were updated to be in line with the 2021 WHO classification criteria of CNS tumors and used these diagnoses for subsequent analysis. The CNS WHO grading biomarker *CDKN2A*/*B* homozygous deletion was detected in 11 *IDH*-mutant astrocytic gliomas (two primary tumors and 9 recurrences) and one recurrent oligodendroglioma, and hence the tumors were designated as CNS WHO grade 4 and 3, respectively as indicated [1, 40]. The remaining astrocytomas and oligodendrogliomas with retained *CDKN2A/B* kept their histological grade.

Based on the 2021 WHO criteria, the 79 tumors were reclassified into 27 oligodendrogliomas (16 CNS WHO grade 2 and 11 CNS WHO grade 3) and 52 *IDH*-mutant astrocytomas (16 CNS WHO grade 2, 19 CNS WHO grade 3, 17 CNS WHO grade 4), Additional file 1: Figure S1A. The vast majority of the primary oligodendrogliomas (83%, 10 out of 12), were of CNS WHO grade 2 and 60% of these progressed to CNS WHO grade 3 at first recurrence (Additional file 1: Figure S1B). Similarly, a high proportion of the CNS WHO grade 2 and 3 primary astrocytomas (78%, 18 out of 23) recurred with malignant transformation into higher grades.

### DNA methylation patterns reflect tumor progression

**Fig. 1:**
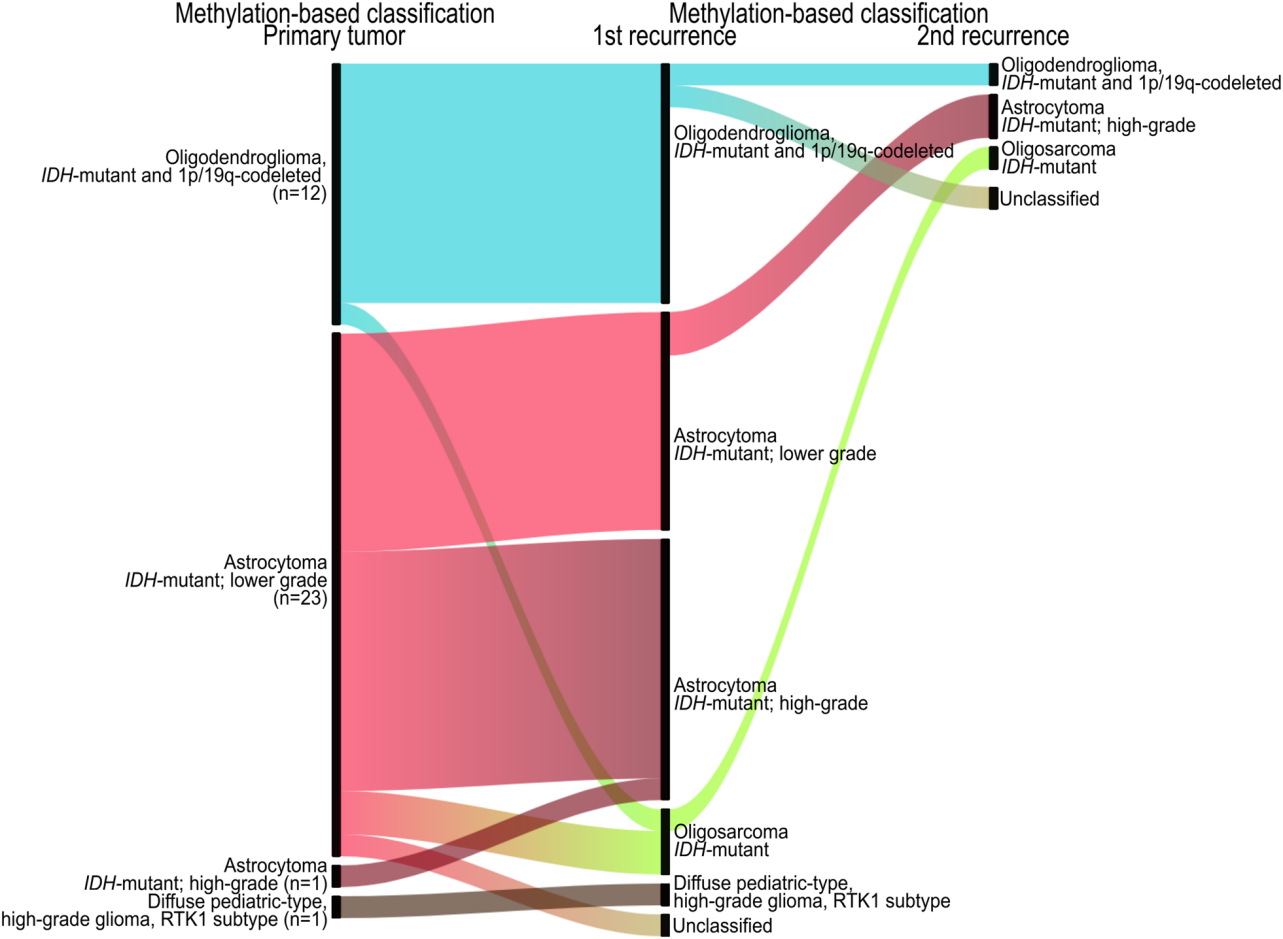
Diagnostics by DNA methylation profiling reflect tumor progression. Molecular classification of the tumor samples by methylation profiling indicating methylation subclasses with the highest predicted calibrated scores according to MNP classifier [19]

We studied how genome-wide DNA methylation patterns vary during *IDH*-mutant glioma progression. Clustering analysis of SNPs verified the patient identity of all tumor tissue specimens (Additional file 2: Figure S2A). Tumor cell content, based on the methylation profiles of the tumors, did not differ significantly between the primary and the recurrent tumors (Additional file 2: Figure S2B). To provide an overview of methylation-based classification along tumor recurrence, we applied the MNP classifier (version 12.5) [19] and examined the predicted top methylation subclasses (Fig. 1). Out of the 79 tumor cases, 75% (n = 59) were successfully classified into methylation subclasses (calibrated score ≥ 0.90) by the MNP classifier (Additional file 5: Table S1 and Additional file 2: Figure S2C). Tumor purity was overall higher in successfully classified cases compared to cases with calibrated scores below the 0.90 threshold (Additional file 2: Figure S2D), and tumor recurrences tended to receive lower calibrated scores than the primary tumors. The lower classification scores than the 0.90 threshold (as low as 0.50) could still, however, provide valid diagnostic classifications in many of the recurrent cases in our cohort as we also previously demonstrated for the primary tumors [10].

**Fig. 2:**
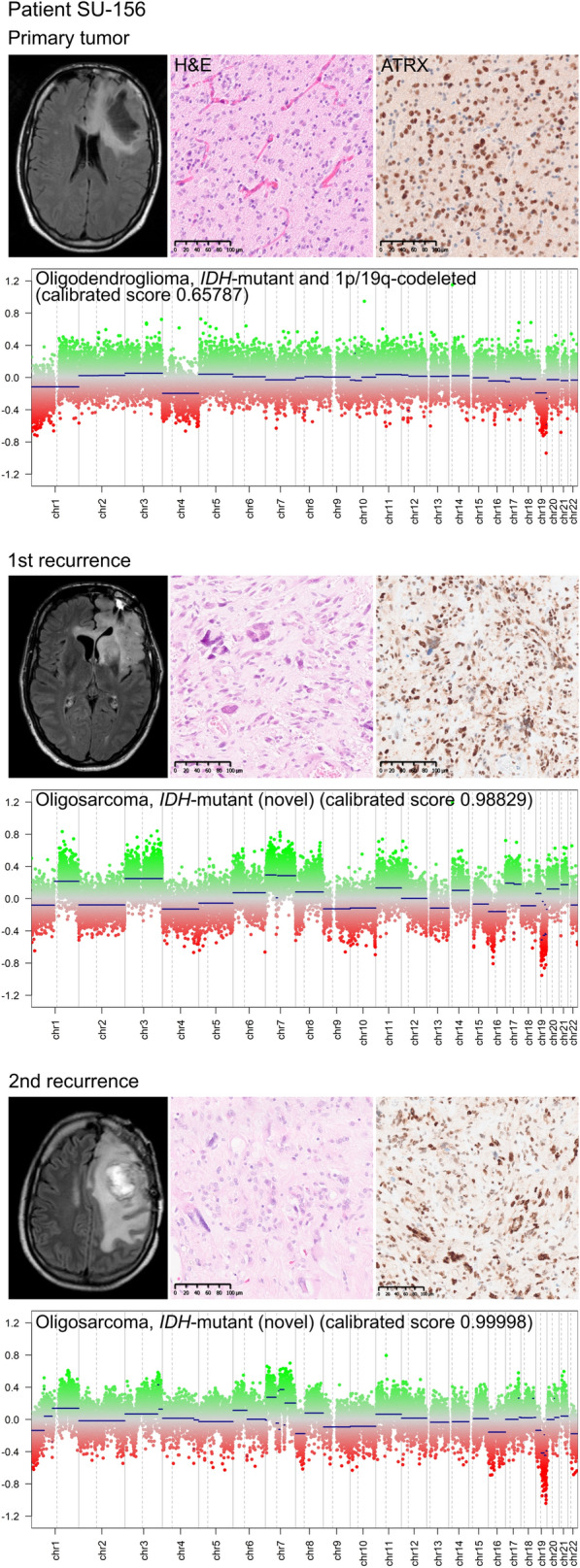
Examination of an oligodendroglioma case with differential methylation diagnosis. Patient SU-156 developed two local recurrences in the frontal lobe as shown by MRI scans. The primary tumor was subclassified as oligodendroglioma, *IDH*-mutant and 1p/19q-codeleted by DNA methylation profiling, whereas the tumor recurrences were assigned to the novel oligosarcoma, *IDH*-mutant subclass. The primary tumor showed typical oligodendroglioma morphology by hematoxylin and eosin (H&E) stains whereas the recurrent tumors had sacromatous components. Nuclear positivity of ATRX was detected in all the three tumor samples. Copy number alteration profiles, retrieved from the methylation array data, indicated hyperdiploidy in the tumor recurrences. Scale bars: 100 µm

The primary oligodendroglioma CNS WHO grade 2 and 3 cases (n = 12) were methylation-based classified as the oligodendroglioma, *IDH*-mutant and 1p/19q-codeleted subclass and 83% (10 out of 12) maintained the methylation subclass at recurrence (Fig. 1). In one of the oligodendroglioma cases with two tumor recurrences (SU-156), the classifier predicted the primary CNS WHO grade 2 tumor as an oligodendroglioma, *IDH*-mutant and 1p/19q-codeleted subclass (calibrated score 0.65787), and the paired CNS WHO grade 3 recurrences were assigned to the novel oligosarcoma, *IDH*-mutant subclass (calibrated scores > 0.90, Fig. 2). We re-evaluated the clinical, histological and molecular data previously acquired for this patient. The tumors presented IDH1 (R132H) mutations detected by IHC and Sanger sequencing (performed for the primary tumor only) [10] and showed nuclear immunopositivity of ATRX in consistence with the 2021 WHO classification criteria for the diagnosis of oligodendroglioma, *IDH-*mutant and 1p/19q-codeleted. CNA profiles of the tumor recurrences displayed an increased load of copy number variations that resembled hyperdiploidy, where 1p/19q-codeletions with concurrent polysomi were detected in these cases and also detected by FISH (data not shown). Histopathological observation revealed areas with sarcomatous features present in the recurrent tumors whereas the primary tumor mainly showed classical oligodendroglial histomorphology. The patient had a rapid deterioration with a shorter survival time (~ 6 years from primary surgery) than the median survival time of oligodendroglioma (~ 16 years) [23]. Altogether, these reevaluations supported the classifier’s prediction of the tumors.

Of the 25 primary *IDH*-mutant astrocytomas CNS WHO grade 2–4, 23 were subclassified as astrocytoma, *IDH*-mutant of lower-grade (calibrated scores 0.50–0.99) and the proportion of cases predicted as astrocytomas, *IDH*-mutant of high-grade, increased at recurrence (Fig. 1). Remarkably, three CNS WHO grade 4 astrocytomas (SU-30, SU-139 and SU-168) were assigned to the lower-grade astrocytoma *IDH*-mutant methylation subclass (calibrated scores 0.55–0.99). In two of these cases (SU-139 and SU-168, both primary tumors), homozygous deletions of *CDKN2A*/*B* were detected on their CNA profiles inferred from the methylation array data. These CNS WHO grade 4 astrocytomas patients experienced a tumor recurrence between 3 and 4 years after primary surgery and had an overall survival of ~ 5 years.

**Fig. 3:**
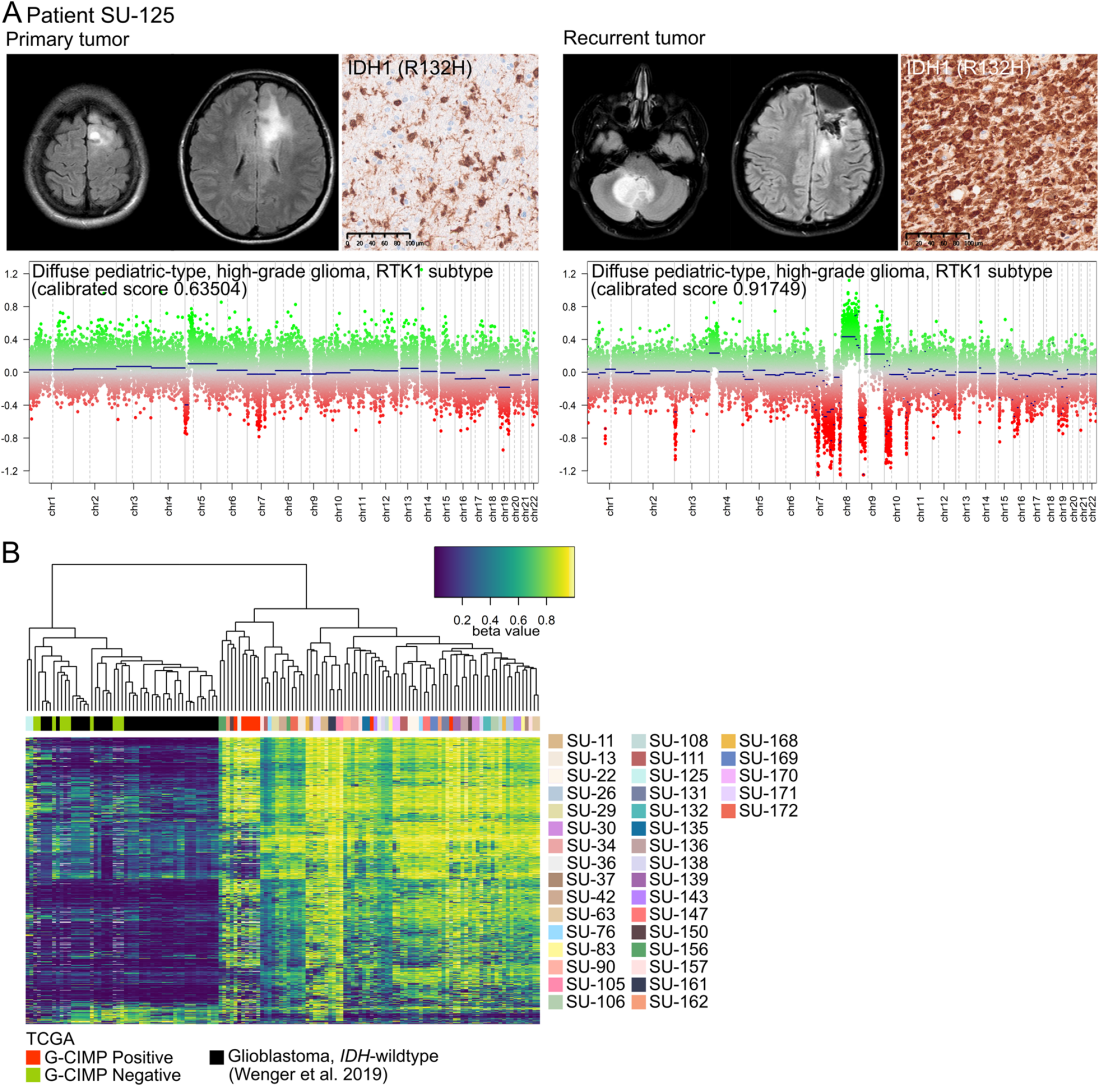
Examination of an *IDH*-mutant astrocytoma case with differential methylation diagnosis. **A** Patient SU-125 developed a distant tumor recurrence (from frontal lobe to cerebellum) as shown by MRI scans. The primary tumor and paired recurrence were assigned to the *IDH*-wildtype subclass diffuse pediatric-type high-grade glioma, RTK1 subtype. Both tumors harbored IDH1 (R132) mutations by immunohistochemistry, **B** and exhibited a negative glioma CpG island methylator phenotype (G-CIMP; green) by hierarchical clustering of the *IDH*-mutant glioma samples with The Cancer Genome Atlas (TCGA) glioma samples [31] and glioblastoma, *IDH*-wildtype samples from Wenger et al. with a G-CIMP Negative signature (black) [38]. The tumor samples are color-coded by patient identity. Scale bars: 100 µm

We detected one astrocytoma, *IDH*-mutant case (SU-125) where the primary CNS WHO grade 3 tumor and paired CNS WHO grade 4 recurrence were classified as diffuse pediatric-type high-grade glioma, RTK1 subtype (calibrated scores 0.63514 and 0.91749, respectively), which is a subclass comprised of *IDH*-wildtype diffuse gliomas with malignant histomorphology that can occur in children and young adults [19]. After reevaluation of this case, we observed that the patient experienced a distant tumor recurrence, where the primary tumor was in the frontal lobe while the recurrence was in the cerebellum, see Fig. 3A. Furthermore, IDH1 (R132H) mutations were detected in the tumors by IHC and Sanger sequencing (performed for the primary tumor only) [10]. The tumors displayed a G-CIMP negative profile (Fig. 3B), which is associated with *IDH*-mutant gliomas with poor prognosis [9, 21]. This was a relatively young patient (19 years at initial diagnosis) and the patient had a short survival time (~ 1 year) in comparison to the typical survival time of *IDH*-mutant gliomas. This is in agreement with the poor prognosis of G-CIMP negative, usually associated with *IDH*-wildtype gliomas. Two other *IDH*-mutant astrocytomas (SU-29 and SU-83) were classified as the oligosarcoma subclass at recurrence (calibrated scores 0.899 and 0.549, respectively), Additional file 3: Figure S3. In both cases, the tumor recurrences did not harbor 1p/19q codeletions and showed pleomorphic and gemistocytic astrocytomas but no clear morphological patterns of sarcoma were detected.

**Fig. 4:**
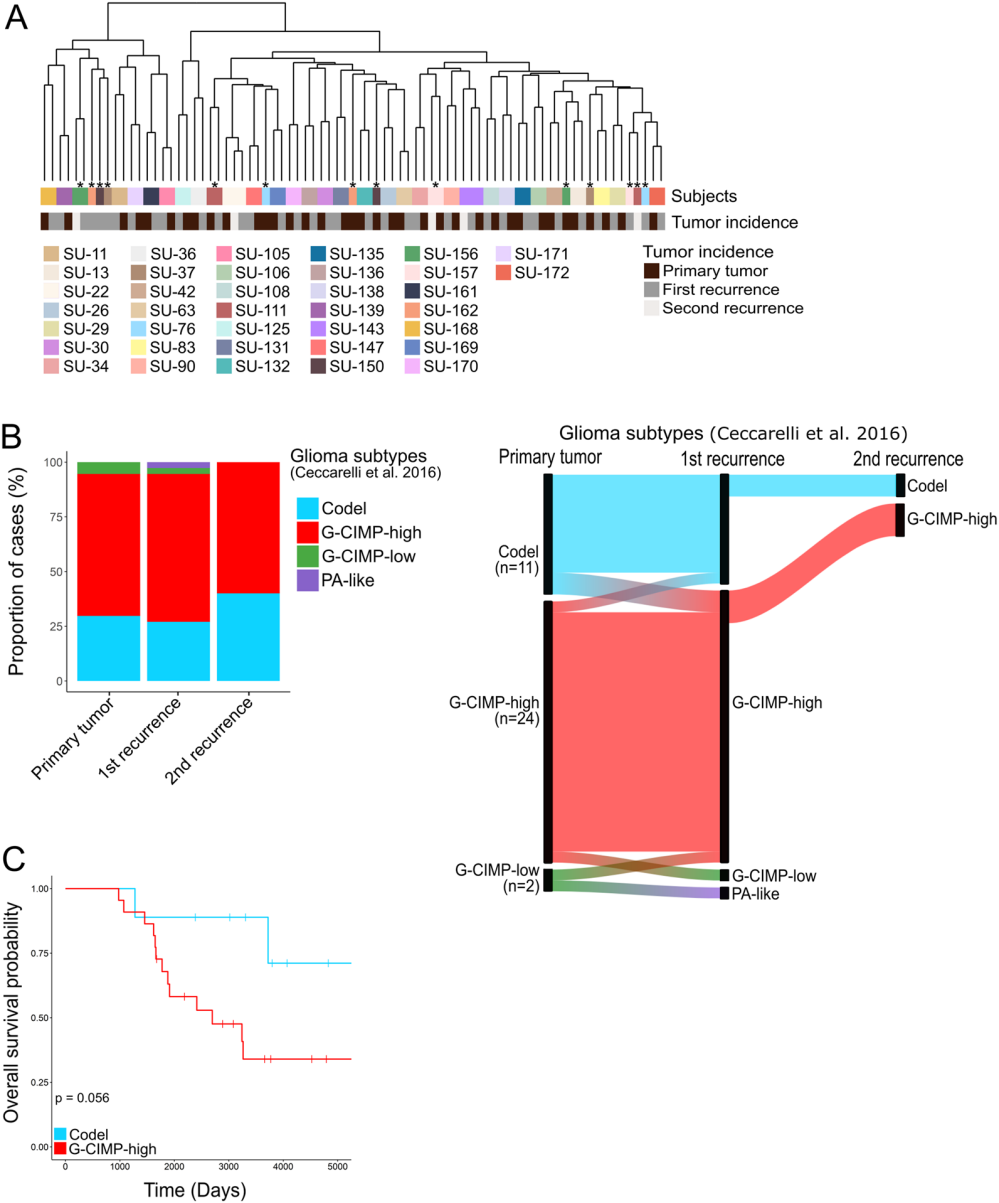
DNA methylation patters remain stable upon recurrence. **A** Unsupervised hierarchical clustering analysis of the top 10 000 most variable CpG sites on the methylation array clusters the tumor samples from each patient together except for seven cases, represented in the figure with an asterisk (*). The tumor samples are color-coded by patient identity and tumor incidence. **B** Proportion of cases based on glioma subtypes from Ceccarelli et al. [7] (left). The G-CIMP-high/low signature of the tumors was mainly maintained during tumor progression (right). **C** Overall survival of patients based on the glioma subtypes. Significance: *p*-value < 0.05

We further examined DNA methylation alterations by unsupervised hierarchical clustering analysis of the top 10 000 most variable CpG sites on the methylation array (Fig. 4A). For 81% of the cases (30 out of 37 patients), tumor samples from the same patient clustered together regardless of the methylation subclass (Additional file 5: Table S1), thus indicating a rather stable DNA methylation landscape during tumor progression. However, for seven patients, the paired primary and recurrent tumors did not group together and five out of these had a shift in methylation subclass; e.g. the primary lower-grade astrocytomas switched to high-grade astrocytomas upon recurrence (n = 4), and in one oligodendroglioma case, the primary tumor progressed to oligosarcoma (Fig. 2).

We then evaluated methylation alterations in relation to G-CIMP since demethylation of G-CIMP has been associated with malignant transformation and recurrence of gliomas [7]. The primary tumors in our cohort were predominantly G-CIMP-high (65%, 24/37) where the vast majority of these (22/24) maintained their G-CIMP-high status at recurrence (Fig. 4B). The overall survival probability in patients with G-CIMP-high tumors (n = 22) was low compared to patients with Codel tumors (n = 9), but this difference was not significant (Fig. 4C). Only one primary G-CIMP-high tumor shifted to G-CIMP-low upon recurrence (SU-150), probably reflecting the malignant transformation of the tumor; the primary CNS WHO grade 3 tumor progressed to CNS WHO grade 4.

### Differential methylation at specific CpG sites accumulates during tumor progression

**Fig. 5:**
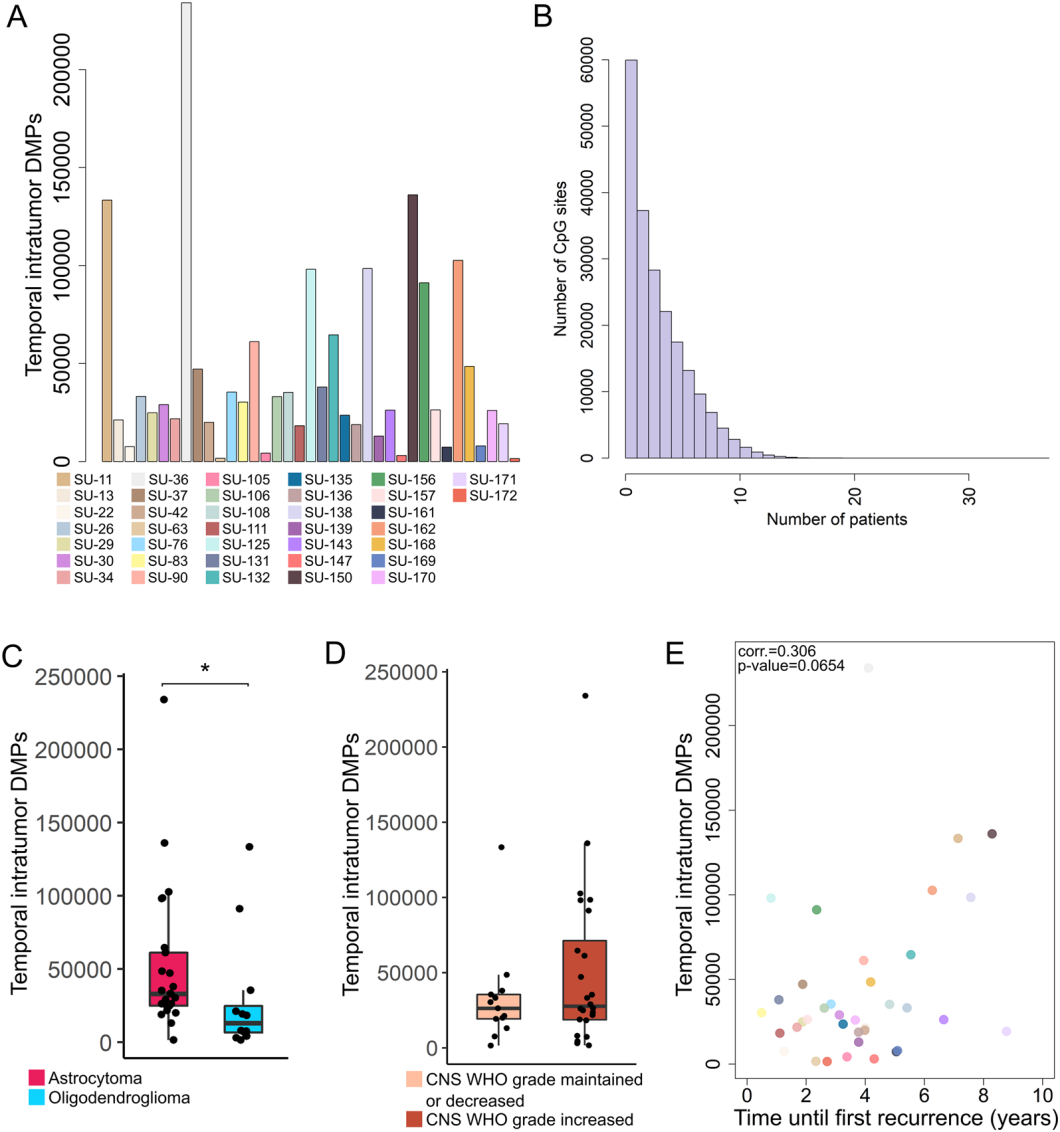
DNA methylation changes at specific CpG sites occur during tumor progression. **A** The number of differentially methylated positions (DMPs) between the primary tumor and first recurrence of individual patient varies between patients, and **B** only few DMPs were shared between most of the patients. **C** Astrocytomas accumulated significantly more DMPs over time compared to oligodendrogliomas. **D** The number of DMPs tended to increase with increasing CNS WHO tumor grade, **E** but DMPs were not associated with the time between the primary tumor and first recurrence. *Denotes significance (*p*-value < 0.05). The tumor samples are color-coded by patient identity, and this applies to **A** and **E** panels

To evaluate DNA methylation changes associated with *IDH*-mutant glioma progression, we analyzed the number of DMPs between paired tumors of individual patients (“temporal intratumor DMPs”) and between tumor groups (i.e. unpaired primary vs. recurrent tumors). The number of temporal intratumor DMPs largely differed between the patients (mean 44,471, max: 232,378, min: 1374, Fig. 5A), however, only a minority of these DMPs were shared between most of the patients (four DMPs were present in 19/37 patients, Fig. 5B, Additional file 4: Figure S4A). In addition, patients with *IDH*-mutant astrocytomas CNS WHO grades 2–4 had significantly more DMPs compared to patients with oligodendrogliomas CNS WHO grade 2–3 (mean DMPs 51,700 and 29,200, respectively, *p*-value = 0.01045, Fig. 5C). There was a trend of increased number of DMPs with malignant tumor progression in comparison to tumors that maintained or decreased in grade over time (Fig. 5D), where astrocytomas usually accumulated more DMPs during malignant progression (Additional file 4: Figure S4B). Postoperative radiotherapy with or without temozolomide resulted in a significantly larger number of CpG site-specific alterations compared to surgery alone (*p*-value < 0.05, Additional file 4: Figure S4C and Additional file 6: Table S2). Furthermore, there was no significant correlation between the number of DMPs and the time until first tumor recurrence (Fig. 5E).

We identified a total number of 35 952 DMPs between the primary and recurrent tumor groups. Only 326 CpG sites had difference in methylation levels of more than 20% between the tumor groups (Additional file 7: Table S3). The majority of these CpG sites (92%, 301/326) were hypomethylated in the recurrent tumors compared to the primary tumors and were enriched in OpenSea regions (Additional file 4: Figure S4D). We further conducted a GSEA on the total number of DMPs identified and found that DMPs were mapped to genes belonging to biological processes associated with the organization of cellular components (organelles and cytoskeletons) and molecular functions related to protein and enzyme binding.

### Tumor progression is associated with alterations in chromosomal copy number and methylation markers

**Fig. 6:**
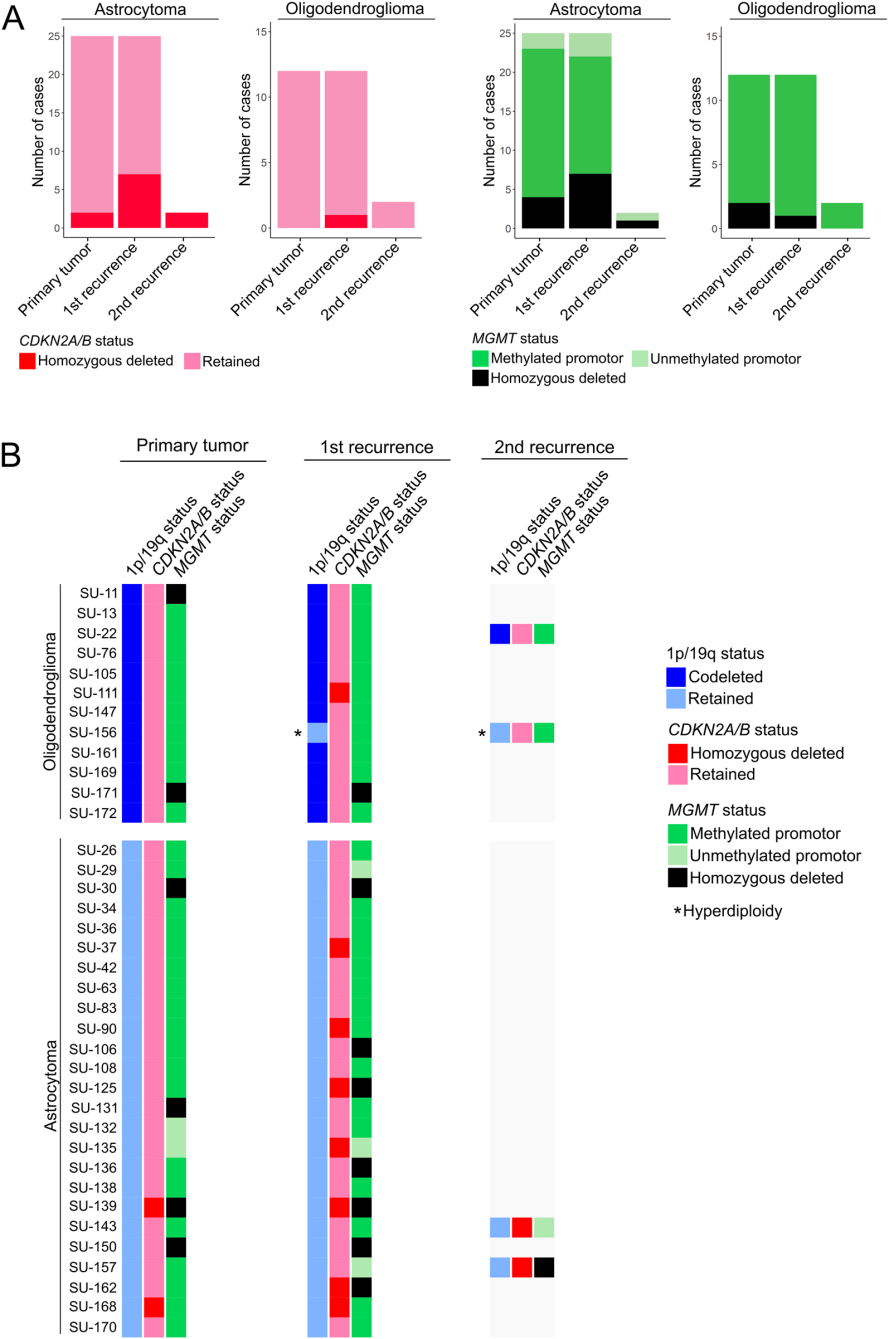
DNA methylation and chromosomal copy number alterations occur at specific markers over time. **A** Proportion of astrocytomas and oligodendrogliomas over time based on *CDKN2A/B* status (left) and *MGMT* promotor methylation status (right). **B** *CDKN2A/B* status and *MGMT* promotor methylation status were stable throughout tumor progression of oligodendrogliomas whereas these markers were commonly altered over time in astrocytomas. 1p/19q-codeleted (blue) or retained (light blue). *CDKN2A/B* homozygous deleted (red) or retained (pink). Methylated *MGMT* promotor (green), unmethylated *MGMT* promotor (light green) and *MGMT* homozygous deleted (black). Tumor cases with indication of hyperdiploidy are represented in the figure with an asterisk (*)

We next explored whether specific genetic and methylation markers were gained/amplified or lost upon *IDH*-mutant glioma progression. The unclassified second recurrence of one of the patients (SU-111) was excluded from the analysis. Two primary astrocytomas had a *CDKN2A/B* homozygous deletion and this alteration was maintained upon recurrence whereas another seven primary astrocytomas acquired *CDKN2A*/*B* homozygous deletions over time (Fig. 6A, B). The *MGMT* promotor was methylated in 19 primary astrocytomas and in three out of these, the *MGMT* promotor was unmethylated upon recurrence. Notably, in five tumors from four astrocytoma patients, *CDKN2A*/*B* homozygous deletions were accompanied with homozygous deletions of *MGMT.* Homozygous deletion of *CDKN2A*/*B* was less common in oligodendrogliomas as only one recurrent tumor presented this alteration in our entire cohort. A methylated *MGMT* promotor was observed in near all primary oligodendrogliomas and *MGMT* remained methylated at recurrence. The deletions of chromosomal arms 1p and 19q were maintained in all oligodendroglioma cases upon recurrence except for one case (SU-156) where the copy number abnormalities in the recurrent tumors were associated with hyperdiploidy (Figs. 2, 6B).

## Discussion

DNA methylation is an epigenetic mechanism that is altered dynamically over the human lifespan and aberrant methylation changes may lead to the development of diseases including cancer [4, 13]. In this study, we shed light on the DNA methylation changes that occur along progression of adult-type diffuse *IDH*-mutant gliomas.

Methylation profiling was able to reflect tumor progression in *IDH*-mutant gliomas. Hierarchical clustering grouped the majority of the tumor samples by patient identity regardless of the methylation subclass, indicating a higher similarity in DNA methylation profiles between the paired samples of individual patients and thus a rather stable DNA methylation over time. Nevertheless, we also detected higher methylation dissimilarities between the paired samples in seven out of 37 cases as these were not grouped together in the cluster. In five out of these seven cases, we further noticed a methylation subclass switch to higher grades, e.g. from lower-grade astrocytoma to high-grade astrocytoma. These changes in DNA methylation pattern could indicate methylation alterations within cells, but it could also reflect the evolution of clones with distinct methylation profiles through tumor progression.

Oligodendrogliomas with sarcomatous phenotypes, so called oligosarcomas, have been reported as tumors originating from initial oligodendrogliomas, *IDH*-mutant and 1p/19q-codeleted or arising de novo [27, 35, 42]. In this study, methylation profiling identified one case (SU-156) of a primary oligodendroglioma progressing as oligosarcoma over time. 1p/19q codeletions were present in the primary tumor but these were retained upon recurrence, which might be explained by the presence of polysomy in the tumors as previously detected [27, 30]. Furthermore, it has been shown that oligosarcomas exhibit a distinct methylation profile that differs from the conventional methylation patterns of oligodendrogliomas [30], which is in line with our findings, as the primary oligodendroglioma did not group together with the oligosarcoma recurrences by hierarchical clustering analysis of the top variable CpG sites on the methylation array. Since IDH1 (R132H) mutations and histomorphological features of oligodendroglioma were preserved upon tumor recurrence, it is tempting to speculate that the tumor evolution of this case was reflected by DNA methylation changes over time.

*CDKN2A/B* homozygous deletion has recently been included as a grading biomarker for *IDH*-mutant astrocytomas in the 2021 WHO classification criteria of CNS tumors [16]. CNA analysis based on the methylation array data revealed 11 out of 52 astrocytic gliomas harboring homozygous *CDKN2A/B* deletions in our cohort, and we therefore reclassified these tumors as CNS WHO grade 4. *CDKN2A/B* homozygous deletions were commonly encountered in the recurrent tumors compared to the primary tumors (9/27 cases with *CDKN2A/B* deletion in recurrent tumors versus 2/25 in the primary tumors), thereby reflecting the frequent clinical observation of malignant progression in these tumors. There were indications of *CDKN2A/B* homozygous deletions occurring in combination with homozygous deletion of *MGMT* upon recurrence, but further examinations in larger cohorts are required to validate this event and the possible prognostic significance of this combined alteration in the clinical setting. Interestingly, two CNS WHO grade 4 astrocytomas harboring *CDKN2A/B* homozygous deletions were classified with the lower-grade astrocytoma subclass by methylation profiling, although one of the samples received a low classification score (< 0.90). The methylation-based classifier does not include CNAs in the classification of a given tumor sample, but it rather examines methylation signatures of the tumor to generate the prediction [5]. Therefore, the fact that molecular CNS WHO grade 4 tumors could be classified as lower-grade tumors by methylation profiling was not so unexpected, and the methylation-based classification would presumably not have led to changes in the clinical diagnosis of the patients. However, it remains to be elucidated the prognostic value of methylation-based lower-grade astrocytomas with or without *CDKN2A/B* deletions concerning survival outcomes of the patients.

Site-specific DNA methylation alterations were also identified during tumor progression. We detected an average of ~ 44 000 temporal intratumor DMPs and only few of them were common between most of the patients. This suggest that the DNA methylation alterations occur randomly during *IDH*-mutant glioma progression as we also detected in primary glioblastoma, *IDH*-wildtype [38] and in pediatric brain tumors [39]. We recently detected a mean number of ~ 17 000 DMPs within individual primary *IDH*-mutant gliomas and further noticed an increase of DMPs with CNS WHO tumor grade [11]. This trend was also observed over time as more DMPs were identified in tumors transitioning to higher CNS WHO grades compared to the less aggressive tumors, i.e. tumors that maintained or decreased their grade during progression. It should be noted that the tumors reclassified with a CNS WHO grade 4 upon recurrence were solely astrocytomas as oligodendrogliomas cannot be graded as grade 4 tumors according to 2021 WHO [16]. The number of DMPs detected was also associated with the glioma subtype as astrocytomas accumulated more DMPs over time than oligodendrogliomas. We observed a significant increase in DNA methylation alterations in patients who received radiotherapy with or without temozolomide compared to surgery alone, indicating that radio(chemo)therapy may induce focal changes in the methylome. However, it is also a possibility that the patients who received radiotherapy alone or in combination with temozolomide had more aggressive tumors which might be associated with more DNA methylation alterations.

Using methylation profiling, a successful molecular classification was achieved for 75% *IDH*-mutant gliomas with the MNP classifier. Tumor recurrences were more likely to be classified with lower calibrated scores than the primary tumors, but these tumors still obtained valid subclass predictions in the majority of the cases. In addition, tumor purity tended to be lower in the tumor recurrences compared to the primary tumors. Of note, we estimated tumor purity based on DNA methylation array data with the R package InfiniumPurify and using the reference cohort of *lower-grade gliomas* from TCGA included in the package, which might not be representative for certain recurrent tumors as these are frequently of higher CNS WHO grades. Nevertheless, we chose this reference cohort as it was the most representative for our cohort where the majority of the tumors were gliomas of lower-grades (i.e. CNS WHO grade 2 and 3). In addition, using the reference cohort of *glioblastoma* (commonly used for high-grade gliomas), resulted in similar tumor purity estimates as the *lower-grade glioma* reference cohort.

This study included a cohort of patients with *IDH*-mutant gliomas that underwent additional surgery for glioma recurrence. As observed from the median overall survival (7 years from initial diagnosis), the patients undergoing surgery for tumor recurrence during the follow-up period may be the more aggressive *IDH*-mutated tumors with worse prognosis. Therefore, it may not be entirely representative for the *IDH*-mutated tumors as a whole, although likely representative for patients developing a relative early recurrence (within 5 years of primary surgery) undergoing reoperation. Finally, in clinical practice the decision to reoperate a patient with tumor recurrence/progression is always an individualized decision, and some patients may therefore have tumor progression but refrained from a second surgery [37].

## Conclusions

Genome-wide DNA methylation patterns remained mostly stable over time in *IDH*-mutant gliomas. We did notice that some tumors gained more DNA methylation alterations during progression, which could reflect methylation alterations within cells, but it could also be the result of the evolution of intratumor subclones with distinct methylation patterns through tumor progression. We also detected site-specific methylation changes in tumors of individual patients over time, and these changes were associated with the glioma subtype.

## Supplementary Information

**Additional file 1: Fig. S1.** **A** Proportion of patients with primary and recurrent astrocytomas and oligodendrogliomas. **B** Sankey diagram over the 2021 WHO diagnoses of the primary tumors (left), first recurrences (middle) and second recurrences (right).

**Additional file 2: Fig. S2.** **A** The patient identity was verified by unsupervised hierarchical clustering of single nucleotide polymorphism (SNP) sites included on the methylation array data. The tumor samples are color-coded according to their patient identity. **B** Tumor purity, estimated based on methylation array data, tended to decrease over time. **C** Sankey diagram with the 2021 WHO diagnoses (left) and methylation-based classification (right). **D** The predicted calibrated scores of the methylation subclasses (y-axis) and tumor purity scores (x-axis). The tumor samples are color-coded according to their predicted methylation subclass with the highest calibrated score. The primary tumors are represented as circles and the recurrent tumors are shown as triangles.

**Additional file 3: Fig. S3.** **A** Patient SU-29 and **B** SU-83 developed a local recurrence as shown by MRI scans. The primary tumors were subclassified as Astrocytoma, *IDH*-mutant by DNA methylation profiling, whereas their tumor recurrence was assigned to the novel oligosarcoma, *IDH*-mutant subclass. The tumor recurrences did not exhibit clear features of sarcomatous patterns on hematoxylin and eosin (H&E). Scale bars: 100 µm.

**Additional file 4: Fig. S4.** **A** A minority of differentially methylated positions (DMPs) in paired tumors were shared between patients with astrocytomas (left) and oligodendrogliomas (right). **B** DMPs tended to increase with malignant transformation of astrocytomas whereas oligodendrogliomas accumulate less DMPs with malignant transformation over time. **C** Patients receiving post-operative radiotherapy (RT) with temozolomide (TMZ) or RT alone showed a significantly larger number of DMPs compared to patients treated with surgery only. **D** DMPs found between tumor groups (primary vs recurrent tumors) were mostly hypomethylated in the recurrent tumors and frequently encountered in opensea regions. *Denotes significance (p-value < 0.05).

**Additional file 5.** Molecular diagnosis of the patients according to 2021 WHO CNS criteria and methylation-based classification.

**Additional file 6.** The number of temporal intratumor differentially methylated positions in relation to *MGMT* status, post-operative treatment modality and time to recurrence.

**Additional file 7.** A list of CpG sites with a difference in methylation levels of more than 20% between tumor groups (primary vs tumor recurrences).

## Data Availability

The datasets generated during the current study are available from the corresponding author on reasonable request.

## Abbreviations

CDKN2A/B: Cyclin-dependent kinase inhibitor 2A/B
CNA: Copy number alteration
CIMP: CpG island methylator phenotype
CpG: Cytosine-phosphate-guanine
DMP: Differentially methylated position
FFPE: Formalin-fixed paraffin-embedded
GSEA: Gene set enrichment analysis
IDH: Isocitrate dehydrogenase
MGMT: O6-methylguanine–DNA methyltransferase
MNP: Molecular neuropathology
TCGA: The Cancer Genome Atlas
