# Supplementary figures and images for: Longitudinal DNA methylation analysis of adult-type IDH-mutant gliomas
Source: Acta Neuropathol Commun. 2023 Feb 4;11:23. doi: 10.1186/s40478-023-01520-1 (PMC9899392; doi:10.1186/s40478-023-01520-1)

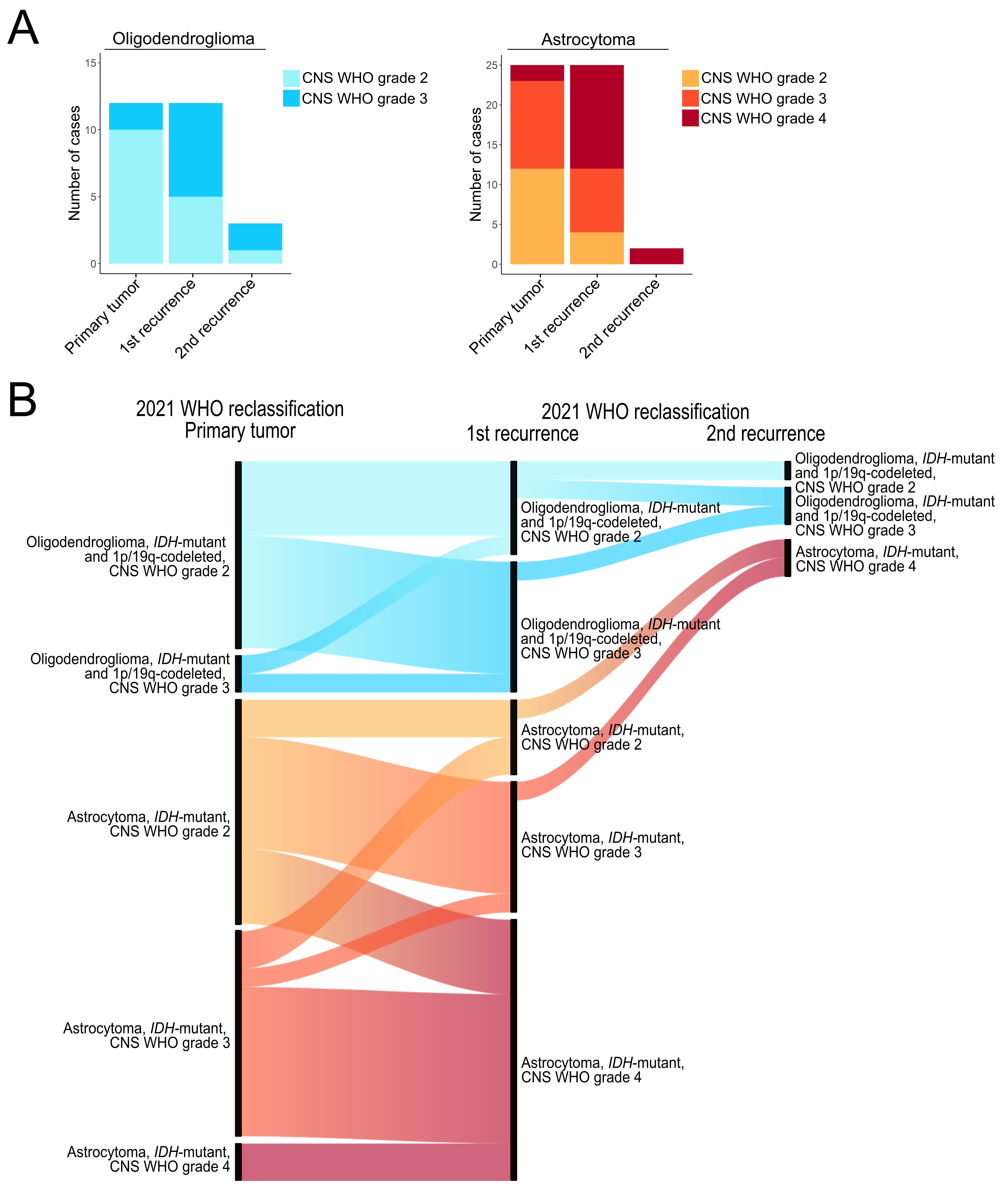

Supplement: Supplementary file 1 — Additional file 1: Fig. S1. A Proportion of patients with primary and recurrent astrocytomas and oligodendrogliomas. B Sankey diagram over the 2021 WHO diagnoses of the primary tumors (left), first recurrences (middle) and second recurrences (right). [file 40478_2023_1520_MOESM1_ESM.tif]

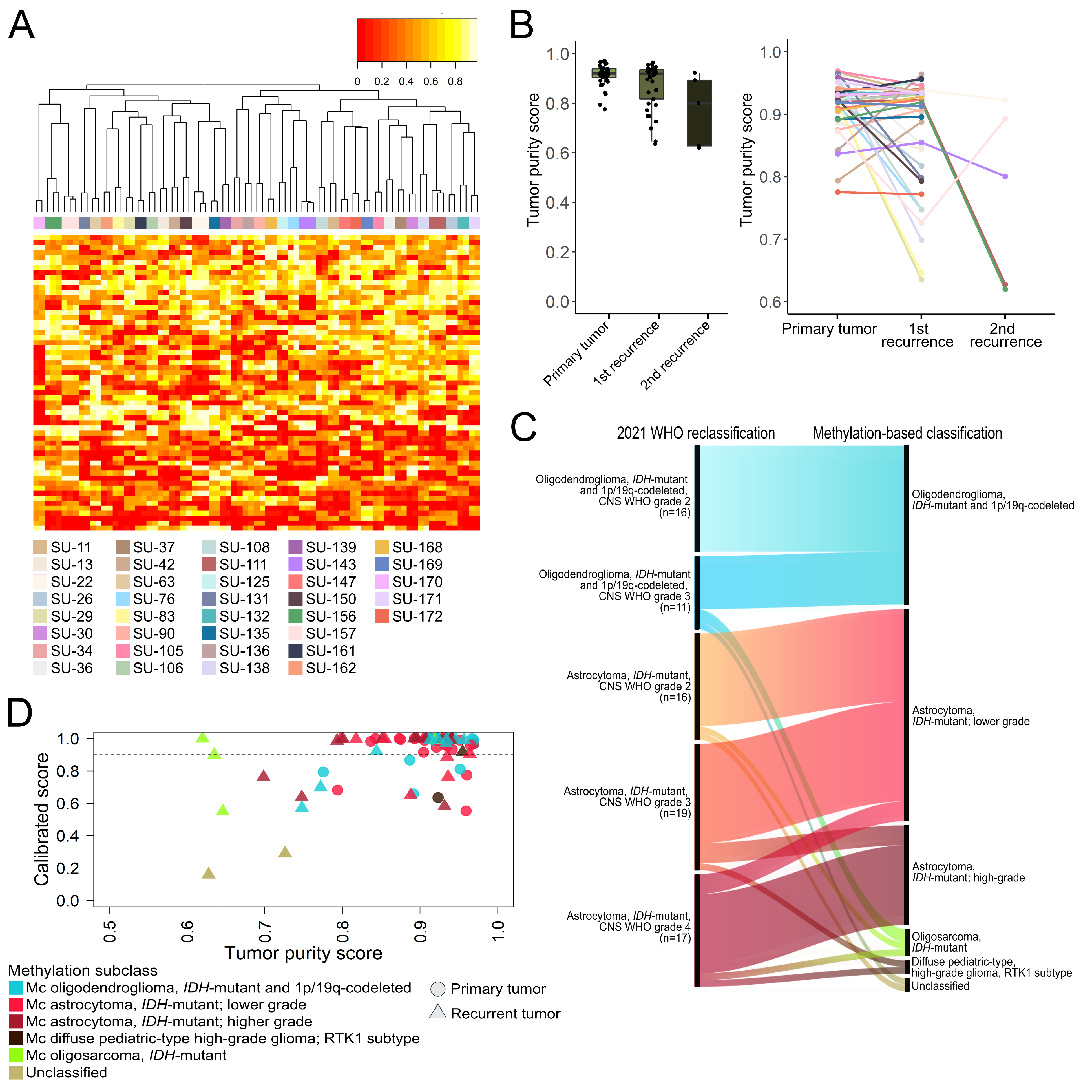

Supplement: Supplementary file 2 — Additional file 2: Fig. S2. A The patient identity was verified by unsupervised hierarchical clustering of single nucleotide polymorphism (SNP) sites included on the methylation array data. The tumor samples are color-coded according to their patient identity. B Tumor purity, estimated based on methylation array data, tended to decrease over time. C Sankey diagram with the 2021 WHO diagnoses (left) and methylation-based classification (right). D The predicted calibrated scores of the methylation subclasses (y-axis) and tumor purity scores (x-axis). The tumor samples are color-coded according to their predicted methylation subclass with the highest calibrated score. The primary tumors are represented as circles and the recurrent tumors are shown as triangles. [file 40478_2023_1520_MOESM2_ESM.tif]

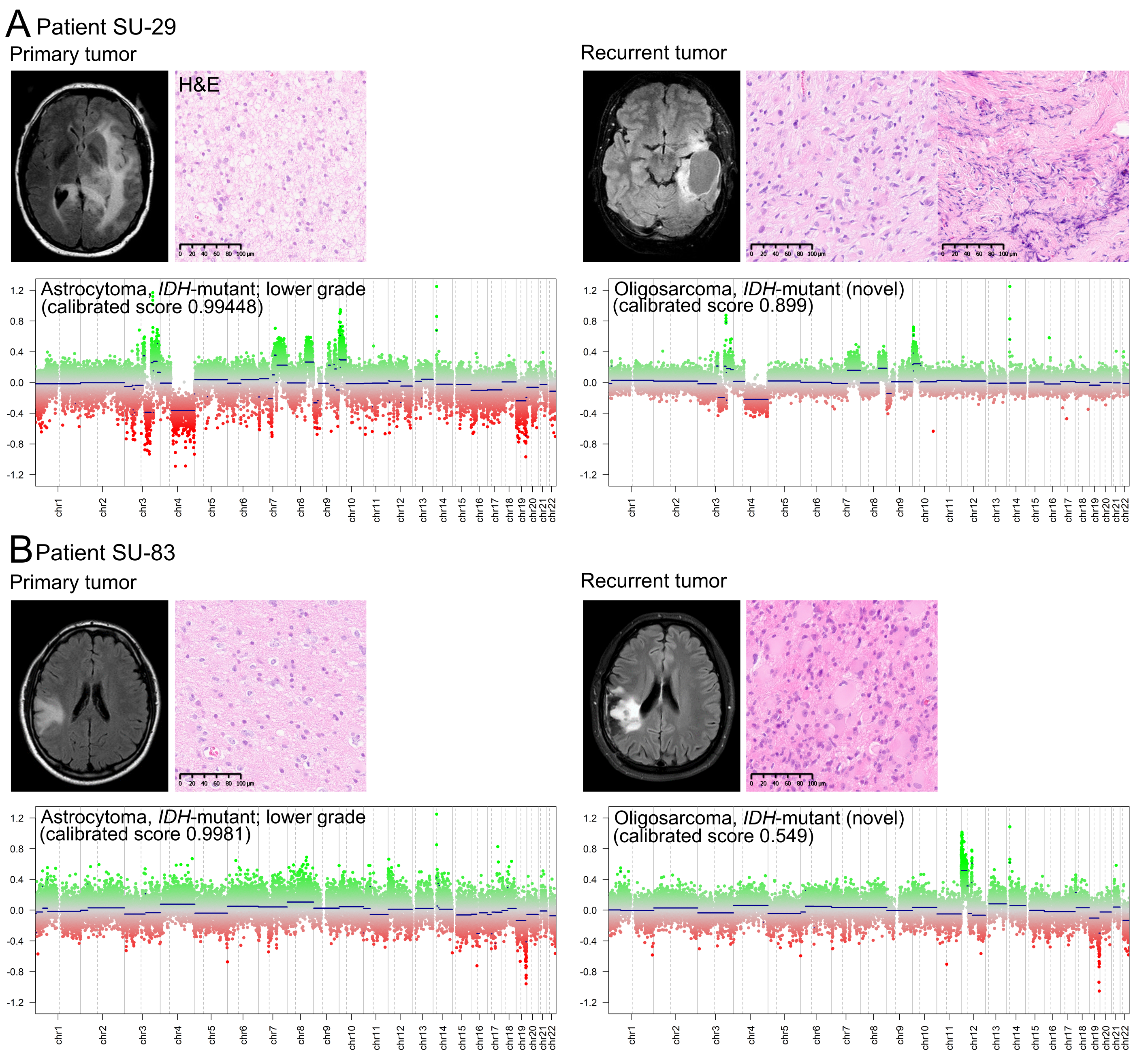

Supplement: Supplementary file 3 — Additional file 3: Fig. S3. A Patient SU-29 and B SU-83 developed a local recurrence as shown by MRI scans. The primary tumors were subclassified as Astrocytoma, IDH-mutant by DNA methylation profiling, whereas their tumor recurrence was assigned to the novel oligosarcoma, IDH-mutant subclass. The tumor recurrences did not exhibit clear features of sarcomatous patterns on hematoxylin and eosin (H&E). Scale bars: 100 µm. [file 40478_2023_1520_MOESM3_ESM.tif]

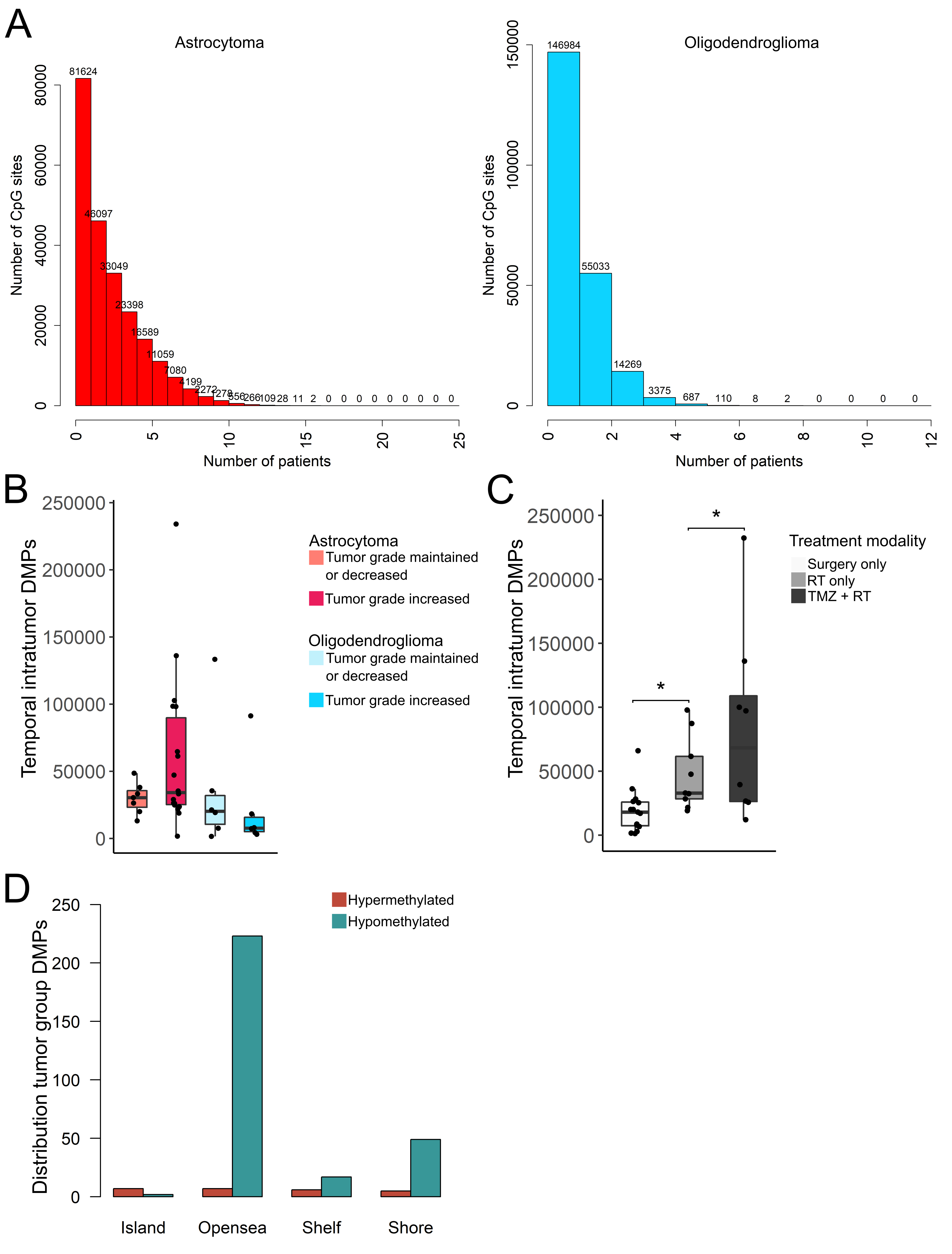

Supplement: Supplementary file 4 — Additional file 4: Fig. S4. A A minority of differentially methylated positions (DMPs) in paired tumors were shared between patients with astrocytomas (left) and oligodendrogliomas (right). B DMPs tended to increase with malignant transformation of astrocytomas whereas oligodendrogliomas accumulate less DMPs with malignant transformation over time. C Patients receiving post-operative radiotherapy (RT) with temozolomide (TMZ) or RT alone showed a significantly larger number of DMPs compared to patients treated with surgery only. D DMPs found between tumor groups (primary vs recurrent tumors) were mostly hypomethylated in the recurrent tumors and frequently encountered in opensea regions. *Denotes significance (p-value < 0.05). [file 40478_2023_1520_MOESM4_ESM.tif]
