# Supplementary material for: Longitudinal DNA methylation analysis of adult-type IDH-mutant gliomas
Source: Acta Neuropathol Commun. 2023 Feb 4;11:23. doi: 10.1186/s40478-023-01520-1 (PMC9899392; doi:10.1186/s40478-023-01520-1)
Supplement: Supplementary file 5 — Additional file 5. Molecular diagnosis of the patients according to 2021 WHO CNS criteria and methylation-based classification. [file 40478_2023_1520_MOESM5_ESM.pdf]

Patient ID	Tumor incidence	Reclassification according to 2021 WHO (Louis DN et al. 2021)	Superfamily (MNP classifier v12.5)	Superfamily calibrated score (MNP classifier v12.5)	Subclass (MNP classifier v12.5)	Subclass calibrated score (MNP classifier v12.5)
SU-11	Primary tumor	Oligodendroglioma, IDH-mutant and 1p/19q-codeleted, CNS WHO grade 3	Adult-type diffuse gliomas	0.99968	Oligodendroglioma, IDH-mutant and 1p/19q-codeleted	0.99912
SU-11	First recurrence	Oligodendroglioma, IDH-mutant and 1p/19q-codeleted, CNS WHO grade 3	Adult-type diffuse gliomas	0.98840	Oligodendroglioma, IDH-mutant and 1p/19q-codeleted	0.97087
SU-13	Primary tumor	Oligodendroglioma, IDH-mutant and 1p/19q-codeleted, CNS WHO grade 2	Adult-type diffuse gliomas	0.96119	Oligodendroglioma, IDH-mutant and 1p/19q-codeleted	0.86616
SU-13	First recurrence	Oligodendroglioma, IDH-mutant and 1p/19q-codeleted, CNS WHO grade 2	Adult-type diffuse gliomas	0.99034	Oligodendroglioma, IDH-mutant and 1p/19q-codeleted	0.92121
SU-22	Primary tumor	Oligodendroglioma, IDH-mutant and 1p/19q-codeleted, CNS WHO grade 2	Adult-type diffuse gliomas	0.99996	Oligodendroglioma, IDH-mutant and 1p/19q-codeleted	0.99970
SU-22	First recurrence	Oligodendroglioma, IDH-mutant and 1p/19q-codeleted, CNS WHO grade 2	Adult-type diffuse gliomas	0.99999	Oligodendroglioma, IDH-mutant and 1p/19q-codeleted	0.99206
SU-22	Second recurrence	Oligodendroglioma, IDH-mutant and 1p/19q-codeleted, CNS WHO grade 2	Adult-type diffuse gliomas	0.99991	Oligodendroglioma, IDH-mutant and 1p/19q-codeleted	0.99956
SU-26	Primary tumor	Astrocytoma, IDH-mutant, CNS WHO grade 3	Adult-type diffuse gliomas	0.99987	Astrocytoma, IDH-mutant; lower grade	0.99747
SU-26	First recurrence	Astrocytoma, IDH-mutant, CNS WHO grade 3	Adult-type diffuse gliomas	0.99938	Astrocytoma, IDH-mutant; lower grade	0.99571
SU-29	Primary tumor	Astrocytoma, IDH-mutant, CNS WHO grade 3	Adult-type diffuse gliomas	0.99974	Astrocytoma, IDH-mutant; lower grade	0.99448
SU-29	First recurrence	Astrocytoma, IDH-mutant, CNS WHO grade 4	Adult-type diffuse gliomas	0.98224	Oligosarcoma, IDH-mutant (novel)	0.89934
SU-30	Primary tumor	Astrocytoma, IDH-mutant, CNS WHO grade 2	Adult-type diffuse gliomas	0.99980	Astrocytoma, IDH-mutant; lower grade	0.94501
SU-30	First recurrence	Astrocytoma, IDH-mutant, CNS WHO grade 4	Adult-type diffuse gliomas	0.99748	Astrocytoma, IDH-mutant; lower grade	0.76447
SU-34	Primary tumor	Astrocytoma, IDH-mutant, CNS WHO grade 2	Adult-type diffuse gliomas	0.99333	Astrocytoma, IDH-mutant; lower grade	0.91527
SU-34	First recurrence	Astrocytoma, IDH-mutant, CNS WHO grade 3	Adult-type diffuse gliomas	0.99953	Astrocytoma, IDH-mutant; high grade	0.99735
SU-36	Primary tumor	Astrocytoma, IDH-mutant, CNS WHO grade 3	Adult-type diffuse gliomas	0.99789	Astrocytoma, IDH-mutant; high grade	0.96813
SU-36	First recurrence	Astrocytoma, IDH-mutant, CNS WHO grade 4	Adult-type diffuse gliomas	0.99982	Astrocytoma, IDH-mutant; high grade	0.99937
SU-37	Primary tumor	Astrocytoma, IDH-mutant, CNS WHO grade 3	Adult-type diffuse gliomas	0.99915	Astrocytoma, IDH-mutant; lower grade	0.98879
SU-37	First recurrence	Astrocytoma, IDH-mutant, CNS WHO grade 4	Adult-type diffuse gliomas	0.99998	Astrocytoma, IDH-mutant; high grade	0.99995
SU-42	Primary tumor	Astrocytoma, IDH-mutant, CNS WHO grade 2	Adult-type diffuse gliomas	0.96253	Astrocytoma, IDH-mutant; lower grade	0.68127
SU-42	First recurrence	Astrocytoma, IDH-mutant, CNS WHO grade 2	Adult-type diffuse gliomas	0.98897	Astrocytoma, IDH-mutant; lower grade	0.64973
SU-63	Primary tumor	Astrocytoma, IDH-mutant, CNS WHO grade 2	Adult-type diffuse gliomas	0.99996	Astrocytoma, IDH-mutant; lower grade	0.99657
SU-63	First recurrence	Astrocytoma, IDH-mutant, CNS WHO grade 3	Adult-type diffuse gliomas	0.99996	Astrocytoma, IDH-mutant; lower grade	0.99626
SU-76	Primary tumor	Oligodendroglioma, IDH-mutant and 1p/19q-codeleted, CNS WHO grade 2	Adult-type diffuse gliomas	0.99985	Oligodendroglioma, IDH-mutant and 1p/19q-codeleted	0.99763
SU-76	First recurrence	Oligodendroglioma, IDH-mutant and 1p/19q-codeleted, CNS WHO grade 2	Adult-type diffuse gliomas	0.74547	Oligodendroglioma, IDH-mutant and 1p/19q-codeleted	0.57098
SU-83	Primary tumor	Astrocytoma, IDH-mutant, CNS WHO grade 3	Adult-type diffuse gliomas	0.99988	Astrocytoma, IDH-mutant; lower grade	0.99881
SU-83	First recurrence	Astrocytoma, IDH-mutant, CNS WHO grade 2	Adult-type diffuse gliomas	0.98161	Oligosarcoma, IDH-mutant (novel)	0.54915
SU-90	Primary tumor	Astrocytoma, IDH-mutant, CNS WHO grade 2	Adult-type diffuse gliomas	0.99985	Astrocytoma, IDH-mutant; lower grade	0.99425
SU-90	First recurrence	Astrocytoma, IDH-mutant, CNS WHO grade 4	Adult-type diffuse gliomas	0.99998	Astrocytoma, IDH-mutant; high grade	0.99995
SU-105	Primary tumor	Oligodendroglioma, IDH-mutant and 1p/19q-codeleted, CNS WHO grade 2	Adult-type diffuse gliomas	0.99662	Oligodendroglioma, IDH-mutant and 1p/19q-codeleted	0.99101
SU-105	First recurrence	Oligodendroglioma, IDH-mutant and 1p/19q-codeleted, CNS WHO grade 3	Adult-type diffuse gliomas	0.99827	Oligodendroglioma, IDH-mutant and 1p/19q-codeleted	0.99220
SU-106	Primary tumor	Astrocytoma, IDH-mutant, CNS WHO grade 3	Adult-type diffuse gliomas	0.99984	Astrocytoma, IDH-mutant; lower grade	0.99005
SU-106	First recurrence	Astrocytoma, IDH-mutant, CNS WHO grade 4	Adult-type diffuse gliomas	0.99987	Astrocytoma, IDH-mutant; high grade	0.98961
SU-108	Primary tumor	Astrocytoma, IDH-mutant, CNS WHO grade 2	Adult-type diffuse gliomas	0.99957	Astrocytoma, IDH-mutant; lower grade	0.98997
SU-108	First recurrence	Astrocytoma, IDH-mutant, CNS WHO grade 3	Adult-type diffuse gliomas	0.96878	Astrocytoma, IDH-mutant; high grade	0.63741
SU-111	Primary tumor	Oligodendroglioma, IDH-mutant and 1p/19q-codeleted, CNS WHO grade 2	Adult-type diffuse gliomas	0.99954	Oligodendroglioma, IDH-mutant and 1p/19q-codeleted	0.98687
SU-111	First recurrence	Oligodendroglioma, IDH-mutant and 1p/19q-codeleted, CNS WHO grade 3	Adult-type diffuse gliomas	0.99948	Oligodendroglioma, IDH-mutant and 1p/19q-codeleted	0.99206
SU-111	Second recurrence	Oligodendroglioma, IDH-mutant and 1p/19q-codeleted, CNS WHO grade 3	Adult-type diffuse gliomas	0.34411	Unclassified	-
SU-125	Primary tumor	Astrocytoma, IDH-mutant, CNS WHO grade 3	Pediatric-type diffuse high-grade gliomas	0.67510	Diffuse pediatric-type, high-grade glioma, RTK1 subtype, subclass A (novel)	0.63504
SU-125	First recurrence	Astrocytoma, IDH-mutant, CNS WHO grade 4	Pediatric-type diffuse high-grade gliomas	0.98103	Diffuse pediatric-type, high-grade glioma, RTK1 subtype, subclass A (novel)	0.91749
SU-131	Primary tumor	Astrocytoma, IDH-mutant, CNS WHO grade 3	Adult-type diffuse gliomas	0.99949	Astrocytoma, IDH-mutant; lower grade	0.95769
SU-131	First recurrence	Astrocytoma, IDH-mutant, CNS WHO grade 2	Adult-type diffuse gliomas	0.99995	Astrocytoma, IDH-mutant; lower grade	0.99959
SU-132	Primary tumor	Astrocytoma, IDH-mutant, CNS WHO grade 2	Adult-type diffuse gliomas	0.98970	Astrocytoma, IDH-mutant; lower grade	0.95443
SU-132	First recurrence	Astrocytoma, IDH-mutant, CNS WHO grade 3	Adult-type diffuse gliomas	0.99996	Astrocytoma, IDH-mutant; lower grade	0.99947
SU-135	Primary tumor	Astrocytoma, IDH-mutant, CNS WHO grade 3	Adult-type diffuse gliomas	0.99999	Astrocytoma, IDH-mutant; lower grade	0.99994
SU-135	First recurrence	Astrocytoma, IDH-mutant, CNS WHO grade 4	Adult-type diffuse gliomas	0.99998	Astrocytoma, IDH-mutant; high grade	0.99980
SU-136	Primary tumor	Astrocytoma, IDH-mutant, CNS WHO grade 2	Adult-type diffuse gliomas	0.99998	Astrocytoma, IDH-mutant; lower grade	0.99976
SU-136	First recurrence	Astrocytoma, IDH-mutant, CNS WHO grade 3	Adult-type diffuse gliomas	0.99789	Astrocytoma, IDH-mutant; lower grade	0.90586
SU-138	Primary tumor	Astrocytoma, IDH-mutant, CNS WHO grade 3	Adult-type diffuse gliomas	0.98098	Astrocytoma, IDH-mutant; lower grade	0.77469
SU-138	First recurrence	Astrocytoma, IDH-mutant, CNS WHO grade 4	Adult-type diffuse gliomas	0.98500	Astrocytoma, IDH-mutant; high grade	0.76158
SU-139	Primary tumor	Astrocytoma, IDH-mutant, CNS WHO grade 4	Adult-type diffuse gliomas	0.99629	Astrocytoma, IDH-mutant; lower grade	0.55306
SU-139	First recurrence	Astrocytoma, IDH-mutant, CNS WHO grade 4	Adult-type diffuse gliomas	0.99837	Astrocytoma, IDH-mutant; high grade	0.58120
SU-143	Primary tumor	Astrocytoma, IDH-mutant, CNS WHO grade 2	Adult-type diffuse gliomas	0.99955	Astrocytoma, IDH-mutant; lower grade	0.98216
SU-143	First recurrence	Astrocytoma, IDH-mutant, CNS WHO grade 3	Adult-type diffuse gliomas	0.99963	Astrocytoma, IDH-mutant; lower grade	0.99802
SU-143	Second recurrence	Astrocytoma, IDH-mutant, CNS WHO grade 4	Adult-type diffuse gliomas	0.99986	Astrocytoma, IDH-mutant; high grade	0.99716
SU-147	Primary tumor	Oligodendroglioma, IDH-mutant and 1p/19q-codeleted, CNS WHO grade 2	Adult-type diffuse gliomas	0.99998	Oligodendroglioma, IDH-mutant and 1p/19q-codeleted	0.99990
SU-147	First recurrence	Oligodendroglioma, IDH-mutant and 1p/19q-codeleted, CNS WHO grade 3	Adult-type diffuse gliomas	0.99998	Oligodendroglioma, IDH-mutant and 1p/19q-codeleted	0.99992
SU-150	Primary tumor	Astrocytoma, IDH-mutant, CNS WHO grade 3	Adult-type diffuse gliomas	0.99824	Astrocytoma, IDH-mutant; lower grade	0.98658
SU-150	First recurrence	Astrocytoma, IDH-mutant, CNS WHO grade 4	Adult-type diffuse gliomas	0.99402	Astrocytoma, IDH-mutant; high grade	0.98647
SU-156	Primary tumor	Oligodendroglioma, IDH-mutant and 1p/19q-codeleted, CNS WHO grade 2	Adult-type diffuse gliomas	0.91024	Oligodendroglioma, IDH-mutant and 1p/19q-codeleted	0.65787
SU-156	First recurrence	Oligodendroglioma, IDH-mutant and 1p/19q-codeleted, CNS WHO grade 3	Adult-type diffuse gliomas	0.99971	Oligosarcoma, IDH-mutant (novel)	0.98829
SU-156	Second recurrence	Oligodendroglioma, IDH-mutant and 1p/19q-codeleted, CNS WHO grade 3	Adult-type diffuse gliomas	0.99999	Oligosarcoma, IDH-mutant (novel)	0.99998
SU-157	Primary tumor	Astrocytoma, IDH-mutant, CNS WHO grade 2	Adult-type diffuse gliomas	0.99999	Astrocytoma, IDH-mutant; lower grade	0.99996
SU-157	First recurrence	Astrocytoma, IDH-mutant, CNS WHO grade 2	Adult-type diffuse gliomas	0.50163	Unclassified	-
SU-157	Second recurrence	Astrocytoma, IDH-mutant, CNS WHO grade 4	Adult-type diffuse gliomas	0.99998	Astrocytoma, IDH-mutant; high grade	0.99996
SU-161	Primary tumor	Oligodendroglioma, IDH-mutant and 1p/19q-codeleted, CNS WHO grade 2	Adult-type diffuse gliomas	0.99999	Oligodendroglioma, IDH-mutant and 1p/19q-codeleted	0.99998
SU-161	First recurrence	Oligodendroglioma, IDH-mutant and 1p/19q-codeleted, CNS WHO grade 3	Adult-type diffuse gliomas	0.99999	Oligodendroglioma, IDH-mutant and 1p/19q-codeleted	0.99206
SU-162	Primary tumor	Astrocytoma, IDH-mutant, CNS WHO grade 2	Adult-type diffuse gliomas	0.99951	Astrocytoma, IDH-mutant; lower grade	0.93250
SU-162	First recurrence	Astrocytoma, IDH-mutant, CNS WHO grade 4	Adult-type diffuse gliomas	0.99941	Astrocytoma, IDH-mutant; high grade	0.99063
SU-168	Primary tumor	Astrocytoma, IDH-mutant, CNS WHO grade 4	Adult-type diffuse gliomas	0.99949	Astrocytoma, IDH-mutant; lower grade	0.98836
SU-168	First recurrence	Astrocytoma, IDH-mutant, CNS WHO grade 4	Adult-type diffuse gliomas	0.99982	Astrocytoma, IDH-mutant; high grade	0.99909
SU-169	Primary tumor	Oligodendroglioma, IDH-mutant and 1p/19q-codeleted, CNS WHO grade 2	Adult-type diffuse gliomas	0.99998	Oligodendroglioma, IDH-mutant and 1p/19q-codeleted	0.99980
SU-169	First recurrence	Oligodendroglioma, IDH-mutant and 1p/19q-codeleted, CNS WHO grade 3	Adult-type diffuse gliomas	0.99980	Oligodendroglioma, IDH-mutant and 1p/19q-codeleted	0.99206
SU-170	Primary tumor	Astrocytoma, IDH-mutant, CNS WHO grade 2	Adult-type diffuse gliomas	0.99944	Astrocytoma, IDH-mutant; lower grade	0.96258
SU-170	First recurrence	Astrocytoma, IDH-mutant, CNS WHO grade 3	Adult-type diffuse gliomas	0.99693	Astrocytoma, IDH-mutant; lower grade	0.88768
SU-171	Primary tumor	Oligodendroglioma, IDH-mutant and 1p/19q-codeleted, CNS WHO grade 3	Adult-type diffuse gliomas	0.97425	Oligodendroglioma, IDH-mutant and 1p/19q-codeleted	0.81107
SU-171	First recurrence	Oligodendroglioma, IDH-mutant and 1p/19q-codeleted, CNS WHO grade 2	Adult-type diffuse gliomas	0.97592	Oligodendroglioma, IDH-mutant and 1p/19q-codeleted	0.99206
SU-172	Primary tumor	Oligodendroglioma, IDH-mutant and 1p/19q-codeleted, CNS WHO grade 2	Adult-type diffuse gliomas	0.95995	Oligodendroglioma, IDH-mutant and 1p/19q-codeleted	0.79350
SU-172	First recurrence	Oligodendroglioma, IDH-mutant and 1p/19q-codeleted, CNS WHO grade 2	Adult-type diffuse gliomas	0.98110	Oligodendroglioma, IDH-mutant and 1p/19q-codeleted	0.69920

MNP: Molecular neuropathology classifier
