# Supplementary material for: Longitudinal DNA methylation analysis of adult-type IDH-mutant gliomas
Source: Acta Neuropathol Commun. 2023 Feb 4;11:23. doi: 10.1186/s40478-023-01520-1 (PMC9899392; doi:10.1186/s40478-023-01520-1)
Supplement: Supplementary file 6 — Additional file 6. The number of temporal intratumor differentially methylated positions in relation to MGMT status, post-operative treatment modality and time to recurrence. [file 40478_2023_1520_MOESM6_ESM.pdf]

Patient ID	MGMT status (primary tumor)	Temporal intratumor DMPs	Time to first recurrence, years (from first surgery)	Post-operative treatment modality (until first recurrence)	Time to second recurrence, years (from first recurrence)
SU-11	Homozygous deleted	138483	7.1	PCV + RT	
SU-13	Methylated promotor	20096	4	Surgery only	
SU-22	Methylated promotor	8739	1.3	Surgery only	1.6
SU-26	Methylated promotor	33000	5.4	RT only	
SU-29	Methylated promotor	25732	1.9	TMZ + RT	
SU-30	Homozygous deleted	28368	3.1	RT only	
SU-34	Methylated promotor	25448	1.7	Surgery only	
SU-36	Methylated promotor	232378	4.1	TMZ + RT	
SU-37	Methylated promotor	47622	1.9	TMZ only	
SU-42	Methylated promotor	20209	4	Surgery only	
SU-63	Methylated promotor	1583	2.3	Surgery only	
SU-76	Methylated promotor	36783	2.8	PCV only	
SU-83	Methylated promotor	31397	0.5	PCV only	
SU-90	Methylated promotor	61644	3.9	RT only	
SU-105	Methylated promotor	4350	3.4	TMZ only	
SU-106	Methylated promotor	32457	2.6	RT only	
SU-108	Methylated promotor	36256	4.8	Surgery only	
SU-111	Methylated promotor	18106	1.1	Surgery only	1.1
SU-125	Methylated promotor	97262	0.8	TMZ + RT	
SU-131	Homozygous deleted	39537	1.1	TMZ + RT	
SU-132	Unmethylated promotor	66130	5.5	Surgery only	
SU-135	Unmethylated promotor	21711	3.2	RT only	
SU-136	Methylated promotor	17175	3.8	Surgery only	
SU-138	Methylated promotor	100016	7.6	TMZ + RT	
SU-139	Homozygous deleted	12128	3.8	TMZ + RT	
SU-143	Methylated promotor	26642	6.7	TMZ + RT	1.3
SU-147	Methylated promotor	2926	4.3	Surgery only	
SU-150	Homozygous deleted	136094	8.3	TMZ + RT	
SU-156	Methylated promotor	87452	2.4	RT only	0.7
SU-157	Methylated promotor	28300	2	Surgery only	1.3
SU-161	Methylated promotor	6881	5	Surgery only	
SU-162	Methylated promotor	97933	6.3	RT only	
SU-168	Methylated promotor	47779	4.2	RT only	
SU-169	Methylated promotor	7982	5.1	Surgery only	
SU-170	Methylated promotor	26323	3.7	Surgery only	
SU-171	Homozygous deleted	19155	8.8	RT only	
SU-172	Methylated promotor	1374	2.7	Surgery only	

DMPs: Differentially methylated positions

MGMT: O6-methylguanine-DNA methyltransferase

PCV: Procarbazine, lomustine (CCNU) and vincristine

TMZ: Temozolomide

RT: Radiotherapy
