# Supplementary material for: Longitudinal DNA methylation analysis of adult-type IDH-mutant gliomas
Source: Acta Neuropathol Commun. 2023 Feb 4;11:23. doi: 10.1186/s40478-023-01520-1 (PMC9899392; doi:10.1186/s40478-023-01520-1)
Supplement: Supplementary file 7 — Additional file 7. A list of CpG sites with a difference in methylation levels of more than 20% between tumor groups (primary vs tumor recurrences). [file 40478_2023_1520_MOESM7_ESM.pdf]

IlmnID	Delta beta≥0.2	Adjusted p-value (Benjamini-Hochberg)	Genomic region
cg21247263	0.34393424	0.006223352	opensea
cg16897025	0.295245162	0.002030967	opensea
cg07995570	0.291258475	0.011025408	island
cg22401352	0.247369319	0.023962403	opensea
cg06316886	0.235724198	0.035892112	shore
cg05211651	0.232687344	0.004537674	shelf
cg07382920	0.231933329	0.019950666	island
cg06158650	0.231835752	0.018474591	island
cg09128567	0.230804354	0.038565479	island
cg19781881	0.221375943	0.000165468	shelf
cg22468316	0.220713919	7.57167E-05	opensea
cg12578575	0.220368347	0.018587832	opensea
cg03242698	0.218738082	0.03336507	shore
cg15985270	0.217909685	0.001693331	shelf
cg19446838	0.217087796	0.019976387	island
cg03477775	0.212088836	5.25487E-05	shore
cg15394328	0.210643756	0.000539343	opensea
cg15912800	0.203926679	0.038922682	island
cg18235734	0.203618105	0.047560435	island
cg00582574	0.203468354	0.001511741	shore
cg02630628	0.202330704	0.007777774	shelf
cg27664418	0.202210458	0.016922527	opensea
cg25092100	0.202004948	0.001073957	shelf
cg00093436	0.201932281	0.011282357	shore
cg08138407	0.201215856	0.016481495	shelf
cg18098839	-0.200018113	0.028053964	opensea
cg13333117	-0.200034527	0.029008084	shore
cg14462670	-0.200078517	0.017113417	shelf
cg09151742	-0.200142885	0.037144068	shore
cg06818159	-0.20017628	0.00769992	opensea
cg04973925	-0.200262329	0.018528545	opensea
cg13518625	-0.200375415	0.027038769	opensea
cg24400450	-0.200454882	0.03089599	opensea
cg25964253	-0.200470637	0.025813054	opensea
cg04316369	-0.200553423	0.013769885	opensea
cg21765178	-0.200879552	0.01625195	opensea
cg00558916	-0.200900579	0.028695619	opensea
cg17854549	-0.200908909	0.023261311	opensea
cg12217831	-0.201061297	0.027169825	opensea
cg07413917	-0.201150673	0.03598238	opensea
cg10107043	-0.201322848	0.025431645	opensea
cg21541912	-0.201366136	0.035771968	opensea
cg10910629	-0.201366953	0.018792095	opensea
cg04533166	-0.201406935	0.04043106	opensea
cg07793808	-0.201493492	0.029343604	shore
cg26519740	-0.201628009	0.029705689	opensea

cg08967684	-0.201629005	0.04865078	opensea
cg01437816	-0.20163578	0.021037799	opensea
cg01895882	-0.201732652	0.028544385	opensea
cg15539839	-0.201758205	0.016724828	opensea
cg22573230	-0.201788251	0.009807696	opensea
cg22686259	-0.201911212	0.023004156	opensea
cg12750103	-0.202137483	0.031389387	shore
cg25948591	-0.202174353	0.006701945	opensea
cg02329630	-0.202202223	0.008557399	opensea
cg17458659	-0.202281888	0.03369764	opensea
cg26130797	-0.202304085	0.04746693	opensea
cg01114033	-0.20231205	0.042661495	opensea
cg06417611	-0.202317104	0.007613437	opensea
cg09920804	-0.202345	0.007988621	opensea
cg25233709	-0.202504262	0.04854247	opensea
cg02119938	-0.202533334	0.04166761	opensea
cg00103115	-0.202651454	0.017067833	opensea
cg11376687	-0.202685299	0.008782782	opensea
cg23142987	-0.202686484	0.04778031	opensea
cg20907863	-0.202827638	0.004072998	opensea
cg04837157	-0.202866905	0.041511747	opensea
cg13993907	-0.202986449	0.021187897	opensea
cg21712893	-0.203020065	0.012647764	opensea
cg07753506	-0.203100476	0.026042664	opensea
cg27228611	-0.203144031	0.016790503	opensea
cg22987601	-0.203158223	0.004671999	opensea
cg25922329	-0.203245482	0.004047366	opensea
cg00320447	-0.203409709	0.009595604	opensea
cg02509804	-0.203614387	0.02074911	opensea
cg02595856	-0.203637986	0.043569086	opensea
cg26544219	-0.203678679	0.021469585	opensea
cg23590393	-0.203741081	0.041238365	opensea
cg06691105	-0.203823816	0.018035302	opensea
cg07505997	-0.203841339	0.028824786	shore
cg23656110	-0.203958821	0.017552909	opensea
cg01919701	-0.203960631	0.003893261	shore
cg17389237	-0.204185925	0.020614284	opensea
cg00074348	-0.204218302	0.019039089	opensea
cg01536547	-0.204281914	0.006040026	opensea
cg26579909	-0.204300624	0.024450721	opensea
cg06611541	-0.204323453	0.017548114	opensea
cg25432300	-0.204327742	0.030227789	opensea
cg05483176	-0.204383032	0.032953662	opensea
cg08677240	-0.204418605	0.005095311	opensea
cg06660563	-0.204580243	0.015710746	opensea
cg09837116	-0.204613612	0.008884481	shelf
cg16149574	-0.204783565	0.019093589	opensea

cg23072383	-0.204914978	0.026395728	shore
cg10586317	-0.204965167	0.009472141	shore
cg21752624	-0.205021908	0.036661621	opensea
cg09686210	-0.205056753	0.031442304	opensea
cg03254928	-0.205084454	0.02879569	opensea
cg18589513	-0.205165614	0.037892379	shelf
cg22716958	-0.205199795	0.013291671	opensea
cg03097846	-0.205351962	0.031131827	opensea
cg11594147	-0.205731546	0.010271255	opensea
cg06925722	-0.205774234	0.019189998	opensea
cg21140898	-0.205817466	0.042732028	shore
cg24522654	-0.205909781	0.005322385	opensea
cg20911406	-0.205987679	0.004668508	opensea
cg12159314	-0.206136942	0.013116837	shore
cg25160899	-0.206246564	0.004340268	shelf
cg20540629	-0.206335498	0.026754321	opensea
cg10113471	-0.206385965	0.014061564	opensea
cg22517107	-0.206477399	0.011301126	opensea
cg19578660	-0.206483676	0.010884917	opensea
cg07372034	-0.206547773	0.005173403	opensea
cg14270162	-0.206598539	0.022805198	opensea
cg06032906	-0.206705496	0.024894957	opensea
cg08439109	-0.206818249	0.041327073	shore
cg05641535	-0.206858624	0.022073225	shelf
cg15708127	-0.206949829	0.03036469	opensea
cg24321933	-0.206952778	0.021824928	opensea
cg13010797	-0.206996581	0.012228227	shore
cg19721740	-0.207111089	0.004922149	shore
cg19160624	-0.207132929	0.002811357	opensea
cg17608361	-0.207388902	0.009436768	opensea
cg01785398	-0.20747619	0.01539455	opensea
cg09110394	-0.207484875	0.020731716	opensea
cg02887530	-0.207625519	0.024996536	opensea
cg04207218	-0.207838555	0.007848677	opensea
cg13329772	-0.207931455	0.01211637	opensea
cg17070310	-0.208050372	0.041464595	opensea
cg02886578	-0.20863183	0.022823786	opensea
cg19944656	-0.208758456	0.023852774	opensea
cg07934856	-0.208782668	0.015236422	opensea
cg20505924	-0.208817847	0.027500654	shelf
cg12362478	-0.208868505	0.026901538	opensea
cg09555736	-0.208909866	0.033461156	shore
cg07944953	-0.208986499	0.018234983	opensea
cg00287009	-0.209048569	0.007631009	opensea
cg24276374	-0.209137572	0.028142717	opensea
cg25802439	-0.209450691	0.009493711	opensea
cg02558361	-0.209473791	0.036311474	opensea

cg06835459	-0.209594704	0.01603639	opensea
cg20700458	-0.209672461	0.028151117	opensea
cg01280597	-0.209802659	0.012640965	opensea
cg04703696	-0.20997813	0.022013207	shore
cg09106510	-0.210047695	0.002333053	opensea
cg19782880	-0.210400397	0.031341137	opensea
cg18502863	-0.210495123	0.025641078	opensea
cg10910258	-0.210502839	0.013887475	opensea
cg26940328	-0.210544467	0.036191438	shelf
cg13299477	-0.210645917	0.017053283	shore
cg22349442	-0.210997521	0.009697724	opensea
cg06943635	-0.21110743	0.032071175	opensea
cg14507785	-0.211124068	0.015968065	opensea
cg26962843	-0.211240565	0.011754363	opensea
cg00383136	-0.211485593	0.022966945	opensea
cg05891548	-0.211724579	0.031569567	opensea
cg04616534	-0.211969536	0.019635709	opensea
cg17216430	-0.211983359	0.006507425	opensea
cg25834322	-0.212072562	0.036696783	opensea
cg04335293	-0.212089036	0.00269117	shore
cg16365842	-0.212150942	0.034635717	shore
cg15836180	-0.212264147	0.013773362	opensea
cg14015765	-0.212312436	0.047428371	opensea
cg21946699	-0.212409414	0.003563479	opensea
cg17220841	-0.212416032	0.006354139	opensea
cg09872737	-0.212664404	0.01408775	opensea
cg13371976	-0.212704529	0.025644106	shore
cg21491100	-0.212735599	0.010122088	shore
cg05357039	-0.212788291	0.016796472	shore
cg05851594	-0.212925421	0.030403583	opensea
cg05654304	-0.213337911	0.025576041	opensea
cg08003943	-0.213403254	0.014882298	opensea
cg04135393	-0.213794392	0.02443175	opensea
cg25378003	-0.213798881	0.018988724	shore
cg08831072	-0.213858216	0.020537843	opensea
cg27032787	-0.214036254	0.032920978	opensea
cg09650495	-0.214078267	0.023473315	shelf
cg00116911	-0.214466285	0.040815731	opensea
cg19405484	-0.214475174	0.023370602	opensea
cg27171100	-0.21466103	0.022778712	opensea
cg24085344	-0.214828451	0.001080985	opensea
cg15955144	-0.215012266	0.014141695	opensea
cg26387933	-0.215235497	0.024492411	opensea
cg10965494	-0.2153103	0.030448237	opensea
cg10522447	-0.21563246	0.041929311	shore
cg03781748	-0.215722432	0.012991653	opensea
cg22266824	-0.215857231	0.006701652	opensea

cg13262048	-0.216051585	0.011267754	opensea
cg06924670	-0.216139434	0.014633926	opensea
cg26666978	-0.216148528	0.022756994	opensea
cg22274117	-0.216181873	0.019984567	opensea
cg01640590	-0.216297396	0.004886566	opensea
cg07267600	-0.216364057	0.015685255	opensea
cg06850159	-0.216832681	0.021131606	opensea
cg17452615	-0.217097917	0.014127621	shore
cg08715148	-0.217129117	0.014733064	opensea
cg09096787	-0.217890903	0.001351641	shelf
cg04279049	-0.217914665	0.014643116	opensea
cg08746798	-0.217924143	0.012332657	opensea
cg04341486	-0.217980251	0.016005049	opensea
cg14739799	-0.218136763	0.01326158	opensea
cg24550369	-0.218420719	0.011478706	opensea
cg23322868	-0.21844898	0.023658475	shore
cg06502279	-0.218492961	0.024859602	shore
cg22616810	-0.218551868	0.01028392	opensea
cg02626929	-0.218641616	0.010973116	shore
cg11569198	-0.218676954	0.00551487	shore
cg15234492	-0.219096488	0.021408868	shore
cg19186356	-0.219174169	0.032352078	shore
cg13785339	-0.219247891	0.003969191	opensea
cg20251080	-0.219303792	0.019409834	opensea
cg08663999	-0.219391282	0.017741734	opensea
cg06574575	-0.219740501	0.002179379	opensea
cg23774991	-0.21978466	0.00774889	opensea
cg12683236	-0.219904839	0.04445001	opensea
cg01643513	-0.21999085	0.013166088	opensea
cg21453303	-0.220072876	0.017402405	shore
cg01120733	-0.220086899	0.012092194	opensea
cg07351787	-0.220120706	0.022727657	opensea
cg04983296	-0.220259484	0.033150295	shore
cg23661948	-0.220263651	0.000596138	opensea
cg14576502	-0.22053313	0.001291585	opensea
cg11050333	-0.220677449	0.02802716	opensea
cg10848692	-0.220687275	0.019812977	opensea
cg09584225	-0.220872411	0.008278534	opensea
cg22836826	-0.220910067	0.048288807	opensea
cg27525948	-0.220983748	0.018034703	opensea
cg27341060	-0.221346985	0.020744479	opensea
cg10646654	-0.221460118	0.022624432	opensea
cg15281614	-0.221635794	0.026909167	opensea
cg04112626	-0.221817719	0.021612941	shore
cg08285943	-0.222055976	0.005848017	opensea
cg01382649	-0.222075099	0.035491271	shore
cg18067840	-0.222288434	0.029015458	opensea

cg08488177	-0.222397661	0.006261267	opensea
cg17360377	-0.222446662	0.015432724	opensea
cg14781087	-0.222478628	0.031458223	opensea
cg17862561	-0.22305138	0.019875047	opensea
cg12403514	-0.223202387	0.030300669	island
cg01213463	-0.223212767	0.019256761	opensea
cg27650656	-0.223377996	0.004758492	shelf
cg17434676	-0.223638945	0.001101994	shelf
cg17684904	-0.223672309	0.007475662	opensea
cg08620095	-0.223923967	0.019635709	opensea
cg26829529	-0.224136233	0.009738389	opensea
cg14919537	-0.224358074	0.003859108	opensea
cg05385995	-0.225085091	0.036601614	opensea
cg19605110	-0.225162613	0.005048931	opensea
cg02771489	-0.225204603	0.000708329	opensea
cg03362174	-0.225455999	0.003893975	opensea
cg10299383	-0.225944475	0.008146195	shelf
cg14860676	-0.22653434	0.001245214	opensea
cg25755851	-0.226704198	0.001346435	opensea
cg07737873	-0.22721857	0.001999278	opensea
cg20531857	-0.227401602	0.009276086	opensea
cg10830713	-0.228072752	0.025791902	shore
cg22572560	-0.228235991	0.008661249	opensea
cg18089022	-0.228344488	0.002662938	opensea
cg11011354	-0.228530814	0.016560613	opensea
cg20517854	-0.229232367	0.012012309	opensea
cg04084260	-0.229453346	0.012519516	opensea
cg15716637	-0.229515442	0.009241027	opensea
cg18594370	-0.229535597	0.010726897	opensea
cg04905791	-0.230058979	0.004610527	opensea
cg18538668	-0.230206045	0.020120904	opensea
cg20731529	-0.230496024	0.023423804	opensea
cg10466548	-0.230542213	0.00315988	opensea
cg03693203	-0.230821547	0.005583744	opensea
cg07463059	-0.231203426	0.01986166	opensea
cg06204421	-0.231399464	0.00065804	opensea
cg26182406	-0.231441661	0.015855173	shelf
cg09738522	-0.23170115	0.007546096	opensea
cg01544333	-0.233078757	0.009207323	opensea
cg20279192	-0.233093226	0.008073111	opensea
cg27213416	-0.233364332	0.005259368	shelf
cg17177779	-0.233498291	0.015208464	opensea
cg02893644	-0.23409242	0.004318151	opensea
cg13875194	-0.234299094	0.004995751	shore
cg25330366	-0.235267795	0.011154098	opensea
cg24607755	-0.235423305	0.012653987	opensea
cg19415174	-0.23587225	0.00914726	opensea

cg09099349	-0.235972361	0.000689188	opensea
cg02102075	-0.237147405	0.014495452	shore
cg06808725	-0.237201679	0.01358153	opensea
cg17431888	-0.237243402	0.00807429	opensea
cg05048262	-0.237270724	0.003237022	opensea
cg24500927	-0.237671895	0.004104158	opensea
cg04645070	-0.237706222	0.019033096	opensea
cg15529914	-0.237871245	0.014787826	opensea
cg02195719	-0.238203076	0.012200705	shelf
cg13648527	-0.238284152	0.002138462	opensea
cg13369546	-0.23905911	0.006640982	opensea
cg18184107	-0.239124776	0.013128999	shore
cg03363565	-0.240441841	0.014887474	shore
cg08586855	-0.241117947	0.011478706	opensea
cg14944696	-0.241180632	0.003929946	opensea
cg16788099	-0.241818513	0.004395952	shore
cg03529003	-0.242439934	0.030014543	shore
cg20175702	-0.242489382	0.010029305	opensea
cg18114828	-0.243156616	0.002382188	shore
cg20735107	-0.245052357	0.004995751	opensea
cg16166651	-0.245227518	0.011789075	shore
cg17378344	-0.245473967	0.013769885	opensea
cg16584355	-0.246670111	0.001339215	opensea
cg11457267	-0.247144401	0.036070011	shore
cg04988978	-0.2484777	0.015209338	shelf
cg22810226	-0.248799543	0.010588708	opensea
cg02849507	-0.25035512	0.001991606	opensea
cg04592948	-0.250878703	0.009083042	opensea
cg15418499	-0.251080241	0.015229762	opensea
cg15756415	-0.251898074	0.003749492	opensea
cg15433894	-0.252118956	0.020973156	shore
cg15460584	-0.252939036	0.002286011	opensea
cg14915719	-0.253355775	0.026134429	opensea
cg15054077	-0.254893646	0.019919304	island
cg23534492	-0.258220775	0.022075392	shore
cg23683800	-0.259326764	0.001750421	opensea
cg13432945	-0.260526693	0.013239205	opensea
cg21342383	-0.264862652	0.007623521	opensea
cg04212239	-0.267795973	0.003765171	shore
cg23692401	-0.26957636	0.002370863	opensea
cg25615878	-0.269598261	0.001565469	shelf
cg09615501	-0.274650628	0.002957504	shore
cg01764105	-0.276792758	0.005160397	shore
cg23268630	-0.280200342	0.002011854	opensea
cg03339288	-0.281385892	0.015204294	opensea
